# The nuclear GYF protein CD2BP2/U5–52K is required for T cell homeostasis

**DOI:** 10.3389/fimmu.2024.1415839

**Published:** 2024-09-06

**Authors:** Miriam Bertazzon, Almudena Hurtado-Pico, Carlos Plaza-Sirvent, Marc Schuster, Marco Preußner, Benno Kuropka, Fan Liu, Andor Zenon Amandus Kirsten, Xiao Jakob Schmitt, Benjamin König, Miguel Álvaro-Benito, Esam T. Abualrous, Gesa I. Albert, Stefanie Kliche, Florian Heyd, Ingo Schmitz, Christian Freund

**Affiliations:** ^1^ Department of Chemistry and Biochemistry, Protein Biochemistry, Freie Universität Berlin, Berlin, Germany; ^2^ Department of Molecular Immunology, Ruhr-University Bochum, Bochum, Germany; ^3^ Systems-Oriented Immunology and Inflammation Research Group, Helmholtz Centre for Infection Research, Braunschweig, Germany; ^4^ Department of Chemistry and Biochemistry, RNA Biochemistry, Freie Universität Berlin, Berlin, Germany; ^5^ Department of Chemical Biology, Leibniz-Forschungsinstitut für Molekulare Pharmakologie, Berlin, Germany; ^6^ School of Medicine, Universidad Complutense de Madrid, 12 de Octubre Health Research Institute, Madrid, Spain; ^7^ Department of Mathematics and Computer Science, Freie Universität Berlin, Berlin, Germany; ^8^ Department of Physics, Faculty of Science, Ain Shams University, Cairo, Egypt; ^9^ Institute of Molecular and Clinical Immunology, Health Campus Immunology, Infectiology and Inflammation GCI3, Otto-von-Guericke-University, Magdeburg, Germany

**Keywords:** CD2BP2/U5-52K, T cell, spliceosome, Mdm4, p53, exon skipping

## Abstract

The question whether interference with the ubiquitous splicing machinery can lead to cell-type specific perturbation of cellular function is addressed here by T cell specific ablation of the general U5 snRNP assembly factor CD2BP2/U5–52K. This protein defines the family of nuclear GYF domain containing proteins that are ubiquitously expressed in eukaryotes with essential functions ascribed to early embryogenesis and organ function. Abrogating CD2BP2/U5–52K in T cells, allows us to delineate the consequences of splicing machinery interferences for T cell development and function. Increased T cell lymphopenia and T cell death are observed upon depletion of CD2BP2/U5–52K. A substantial increase in exon skipping coincides with the observed defect in the proliferation/differentiation balance in the absence of CD2BP2/U5–52K. Prominently, skipping of exon 7 in Mdm4 is observed, coinciding with upregulation of pro-apoptotic gene expression profiles upon CD2BP2/U5–52K depletion. Furthermore, we observe enhanced sensitivity of naïve T cells compared to memory T cells to changes in CD2BP2/U5–52K levels, indicating that depletion of this general splicing factor leads to modulation of T cell homeostasis. Given the recent structural characterization of the U5 snRNP and the crosslinking mass spectrometry data given here, design of inhibitors of the U5 snRNP conceivably offers new ways to manipulate T cell function in settings of disease.

## Introduction

CD2 Binding Protein 2 (CD2BP2) is a splicing factor firstly identified as part of the U5 small nuclear ribonucleoprotein (snRNP) subunit, and named U5–52K based on its apparent molecular mass (1). The snRNPs subunits, U1, U2, U4/U6 and U5, consisting of proteins and snRNAs, are the building blocks composing the spliceosome. Extensive compositional and conformational rearrangements of the snRNPs accompany the spliceosomal maturation, a pathway ultimately leading to splicing of pre-mRNA (2–4). Although CD2BP2/U5–52K has been clearly found in the 20S U5 snRNP, its localization in the U4/U6.U5 tri-snRNP and in the subsequent spliceosomal complexes was under discussion for several years. Initially, phosphorylated CD2BP2/U5–52K was identified in the U4/U6.U5 tri-snRNP (5, 6), but further investigations, did not observe this protein in the 25S U4/U6.U5 tri-snRNP (7, 8) or in any other spliceosomal stages as 35S U4/U6.U5 tri-snRNP (9), 35S U5 snRNP (10) and spliceosomal complexes (8, 11, 12). Very recently, CD2BP2 was described as an essential scaffolding protein for the maturation and recycling of the U5 snRNP (13, 14). Interestingly, it was found that while acting as an assembly factor interacting with core spliceosomal proteins such as Prp8, the protein has to dissociate from the U5 snRNP for the tri-snRNP to be formed. This is because a more N-terminal “hook” segment of CD2BP2 occludes the site where the essential Dim1/U5–15K protein is to be placed in the tri-snRNP and later splicing complexes. Interestingly, the C-terminal GYF domain of CD2BP2/U5–52K (15–17), aside from binding proline-rich sequences (PRSs) (18), also interacts with Dim1/U5–15K independent of its PRS binding site (19). Thus, CD2BP2 recruits the same protein that eventually competes with its “hook” segment, thus initiating its own dissociation from the U5 snRNP. Seemingly independent of its role in mRNA splicing PRS binding sites for the protein are also contained in the cytoplasmic tail of the T cell adhesion receptor CD2 and led to the independent identification of CD2BP2/U5–52K by yeast-two-hybrid screening (20). However, the physiological role of this interaction remains elusive (20, 21).

At physiological level CD2BP2 has emerged as an essential protein in higher eukaryotes. In *S. cerevisiae* the protein is dispensable, but has been described to act as a linker between chromatin segregation and RNA splicing (22). TEG-1, the homologue of CD2BP2/U5–52K in *C. elegans*, contributes to defining the proliferation/differentiation balance as demonstrated by altered germline sex determination in CD2BP2/U5–52K mutated worms (23, 24). In *D. melanogaster* the CD2BP2/U5–52K family member Holn1, is required for embryogenesis and eye and wing development, and was found to play a role in wound healing by affecting ERK signaling (25). Furthermore, investigations in mice highlighted the essential role of CD2BP2/U5–52K for embryogenesis (26). Constitutive lack of CD2BP2/U5–52K induces growth retardation, delay in ectodermal layer turning during embryogenesis and growth arrest at developmental stage E10.5 with subsequent death and reabsorption of the embryo. Specific ablation of CD2BP2/U5–52K in podocytes, a highly differentiated epithelial cell type forming the outer part of the glomerular filtration barrier, led to proteinuria and fetal kidney failure in mice (26). Cumulatively, available data indicate that CD2BP2/U5–52K and its homologues play a fundamental role early on in multicellular organisms.

Based on the initial findings of CD2BP2/U5–52K as potentially important for T cell function and given that developmental and proliferation processes are well described in this cell type, we decided to investigate the role of CD2BP2/U5–52K in T cells. A well-described developmental path of T cells takes place in the thymus (27). The hematopoietic stem cells (HSC) reach the thymus from the bone marrow where they become early thymic progenitors (ETP). At this stage several differentiation steps take place starting with the double negative stage (DN), where neither CD4 or CD8 co-receptors are expressed, and in which the rearrangement of TCRγ/δ and TCRβ chains occurs. The DN cells mature into double positive (DP) cells where both, CD4 and CD8 receptors are presented on the surface of the cells. At this stage the rearrangement of TCRα proceeds and coincides with the detection of CD69 and CD5 receptors. Subsequently, T cells transit into CD4 and CD8 single positive (SP) cells which then migrate into the peripheral organs (e.g. spleen, blood, lymph nodes) to fulfill their functions. For our investigation we used a CD4-Cre conditional KO mouse model, which ablates the protein in thymocytes at the double positive (DP) state. Protein levels are shown to cease in positive (SP) T cell population and reduced number of SP cells are observed in the thymus. The fewer number of T cells that reach the peripheral lymphoid tissues are relatively enriched in memory T cells compared to naïve T cells. In the absence of CD2BP2/U5–52K, splicing efficiency decreases, resulting in a considerable augmentation of exon skipping. A change in splicing pattern is observed in several transcripts impacting cell proliferation and apoptosis. In particular, the exon 7 skipping in the Mdm4 transcript can be directly related to dysregulation of a p53 driven transcriptional program. Together, our data suggest that depletion of a general splicing factor leads to modulation of T cell homeostasis and they highlight the enhanced sensitivity of naïve T cells compared to memory T cells to changes in CD2BP2/U5–52K levels.

## Material and methods

### Mice and genotyping

CD2BP2/U5–52K^flox/flox^ mice, described in our previous publication (26), were crossed with CD4-Cre mice (28). The mice were bred and maintained under conventional condition in the animal facility “Tierhaus des Bundesinstitut für Risikobewertung” in Berlin (Germany). The offsprings were genotyped for the detection of flox/flox allele and the CD4-Cre gene. The PCR pair of oligonucleotides primers for the floxed allele and the CD4-Cre are listed in the supplemental material (Suppl. list 1). Guidelines of the Landesamt für Gesundheit und Soziales (LaGeSo) in Berlin (Germany) were followed, (permit number TZ 0274/15).

### Lymphocytes isolation and flow cytometry

Thymus, spleen and peripheral lymph nodes were removed from male 8–12 weeks old mice. For isolation of cells, organs were harvested, minced and filtered through a 70 µm nylon strainer. The isolated cells were first resuspended in RPMI-1640 medium supplemented with with 55 mM β-mercaptoethanol, 10 % FBS, 1 % Pen/Strep and 1 % L-Glutamine and subsequently resuspended in PBS supplemented with 0.5 % BSA and 2 mM EDTA at pH 7.2. The splenocytes followed erythrocyte lysis treatments using Ammonium-Chloride-Potassium (ACK) buffer for 1–2 minutes. To remove clumped cells, 40 µm nylon strainers were used before cell count and staining. Cell counting was performed using a TC20 Automated cell counter (Bio-Rad). 1–2x10^6^ cells were plated in 96-well with V-bottom and stained with Zombie Aqua Fixable Viability Kit (BioLegend, #423101) for the detection of live versus dead cells, following the manufacturers protocol. The cells were further stained with conjugated antibody, purchased by BioLegend. The antibodies used were CD4-FITC (clone RMA4.5, #100510), CD8-PE (clone 53–6.7, #100708), CD69-PECy5 (clone H1.3F3, #104509), CD25-BrillantViolet421 (clone 3C7, #12015), CD5-APC (clone 53.7.3, #100625), CD3-APC (clone 17A2, #100235), B220-PECy7 (clone RA3–6B2, #103221), CD62L-PacificBlue (clone MEL14, #104424), and CD44-PECy5 (clone IM7, #103009). After staining, the cells were fixed using BD Cytofix/Cytoperm kit (#554714). Data were acquired using a BD FACS Canto II instrument while FCS express 6 software was used for the analysis (version 6.06.0033). The statistical analysis is precisely described under each flow cytometry graph caption. For viability analysis, 1x10^6^ cells from thymus, spleen and peripheral lymph nodes were isolated. Organ dissection and cell isolation was performed as previously described in this section. Cells were incubated with CD4-PacificBlue (clone RM4–5, #100531, Biolegend), CD8-BV605 (clone 53–6.7, #100743, Biolegend), CD25-PE-Cy7 (clone PC61, #561780, BD Pharmingen) antibodies in FACS buffer (2% BSA in PBS) for 15 minutes at 4°C. Subsequently, cells were washed with FACS buffer and incubated in 7-aminoactinomycin D (#559925, BD Pharmingen) and AnnexinV-APC (#640920, Biolegend) in binding buffer or stained with CellEvent™ Caspase-3/7 Green Flow Cytometry Assay Kit (#C10427, ThermoFisher Scientific) according to manufacturer´s instructions. Samples were acquired using LSR Fortessa (Becton Dickinson & Company) and analyzed by FlowJo software (Becton Dickinson & Company, version 10.8.0).

### Cell sorting

The isolation of the lymphocytes was performed as previously described. The cells were stained with BioLegend dyes: CD4-PacificBlue (clone L3T4, #100428) and CD8-PE-Cy7 (clone 53–6.7, #100721) or with CD4-FITC (clone RMA4.5, #100510), CD8-PE (clone 53–6.7, #100708) conjugated antibodies. Four subpopulations were sorted and isolated from the thymus (CD4^–^-CD8^-^, CD4^+^-CD8^-^, CD4^–^-CD8^+^, CD4^+^-CD8^+^) while only one (CD4^+^-CD8^-^) from the pooled cells derived from spleen and peripheral lymph nodes. The sorting was performed using BD FACS Aria II and BD FACS Aria III cell sorters.

### Proliferation experiments

Lymph node cells and splenocytes were pooled and erythrocytes lysed by treatment with ACK buffer. Subsequently, cells were labeled with 5 µM Carboxyfluorescein succinimidyl ester (CFSE; #65–0850; eBioscience) for 15 min in the dark at 37°C and washed twice with fully supplemented RPMI. 5x10^5^ cells were seeded in a 96-well plate with each condition in duplicates. T cells were activated with 10 µg/ml coated anti-CD3 (clone 145–2C11, #100314, Biolegend). Proliferation, assessed by loss of the dye signal, was analyzed 3 days later by flow cytometry, in which T cells were labeled by CD4-APC (clone RM4–5, #100516, Biolegend) and CD8-PE-Cy7 (clone 53–6.7, #100721).

### Generation of HEK293T- CD2BP2/U5-52K^-/-^ cell line using CRISPR-Cas9 system

For the generation of HEK293T-CD2BP2/U5-52K^-/-^, cell line that does not express CD2BP2/U5-52K protein, the CRISPR-Cas9 method was used. The oligos selected by the online web tool crispr.mit.edu supplied by Zhang Lab MIT, were ligated into pSpCas9(BB) px459 vector (Supplemental material). HEK293T cells were transfected using a mixture of plasmid and PEI (1:4) in Opti-MEM solution. After 48 hours single cell isolation was performed using puromycin treatment: 3 µg/ml of antibiotic for 24 hours. The isolated single cells were then expanded for 1-2 weeks. The quality of the knock out cells was subsequently checked by analyzing CD2BP2/U5-52K protein expression. The cellular lysate of the cells was analyzed using a Western-blot for the detection of CD2BP2/U5-52K protein.

### Western-blot analysis 

To quantify the presence of targeted proteins in the cells, a Western blot was performed. From 2 to 20 µg of cell lysate were supplemented with SDS loading buffer and loaded on a pre-cast acrylamide gel (4-20%, Bio-Rad) with 8 µl of PageRuler Plus ladder (Thermo Fisher Scientific). The gel was placed on a nitrocellulose paper (GE Healthcare) previously soaked in transfer buffer. The blotting was carried out using Mini Trans-Blot (Bio-Rad), following the manufacturer´s protocol. The nitrocellulose membrane was blocked with blocking buffer (5 % w/v of mill powder in TBST) under agitation for 1 hour at room temperature or overnight at 4°C. The membrane was incubated with the antibodies in TBST, under agitation for 1 hour at room temperature or overnight at 4°C. Finally, the membrane was developed using HRP Juice (PJK) and the chemiluminescence was detected using Advancer Fluorescence and ChemoStar (Intas). The membrane was treated with hydrogen peroxide, for 10-20 min at 37°C, and washed intensively with MilliQ water before the new antibody application. For detection of CD2BP2/U5-52K we used an in-house anti-CD2BP2/U5-52K antibody that was prepared as previously described (26), anti-βActin HRP conjugated (abcam #ab20272), secondary anti-rabbit (Jackson and ImmunoResearch, # 111-035-046), anti-FLAG HRP conjugated (Sigma, A8592).

### RNA isolation and reverse transcriptase PCR

RNA was isolated from sorted T cells using RNeasy Plus Mini Kit (QIAGEN, #74136) and ReliaPrep RNA Cell Miniprep System (Promega, #Z6011). In both cases the manufacturers protocols were followed. The RNA extracted from the cells was reverse transcribed for qPCR analysis. 300–500 ng of RNA for each sample were supplemented with 1 µl of Oligo dT, 1 µl of dNTPs (10 mM) up to 14 µl with RNase and DNase free water. The reaction mixture was incubated for 5 min at 65°C, followed by 5 min at 4°C. Finally, 4 µl of First Strand Buffer, 1 µl of DTT (0.1 M) and 200 U of SuperScript III Reverse Transcriptase were added to the reaction mixture, which was then incubated at 50°C for 50 min, followed by 70°C at 15 min.

### qPCR

The RT-qPCR reaction mixture in a 10 µl of volume corresponds to 2 µl of cDNA (1:8 dilution), 2.8 µl water, 0.2 µM forward and reverse primers and 5 µl of SYBR green Master mix. The PCR program was performed in a StepOne Real-time PCR system following the default program, in case of *Arih2* gene detection the temperature for the extension phase was set at 66°C. The data were exported from StepOne software. The primers were chosen using Primers-Blast NCBI browsers and are listed in the supplemental material together with the list of the gene and transcript IDs (Suppl. list 1 and 2). The data were exported from StepOne software and analyzed using Excel (version 2016). The quantification of the alternative spliced exon was performed using the following Equation 1:


(1) $$\Psi =2^{-\left(C_{T}\text{T}-C_{T}C\right)}$$

Where Ψ is the inclusion exon level, which represent the fold change of the alternatively spliced exon (targeted exon) in the consecutive exons in the proximity (control exons). The C_T_ value is the threshold cycle detected for the PCR fragment related to the targeted (C_T_T) and the control $({\text{C}}_{\text{T}}\text{C}$ ) exons.

### RNA sequencing and data processing

The RNA extracted from CD4^+^ isolated T cells from spleen and lymph nodes from three cKO mice (CD2BP2/U5–52K^f/f^ CD4-Cre^tg^) and three control mice (Control: CD2BP2/U5–52K^wt/f^ CD4-Cre^tg^ and CD2BP2/U5–52K^f/f^ CD4-Cre^-^) were sequenced using RNA-seq technology. The RNA quality and integrity were detected on Agilent Technologies 2100 Bioanalyzer (Agilent Technologies; Waldbronn, Germany). The RNA sequencing library was generated from 50 ng total RNA using NEBNext^®^ Single Cell/Low Input RNA Library to following manufactures’s protocols. The libraries were sequenced on Illumina NovaSeq 6000 using NovaSeq 6000 S1 Reagent Kit (300 cycles, paired end run 2x 150 bp) with an average of 8x10^7^ reads per RNA sample. The RNA-seq analysis was performed with Curta system (29). The quality of the fastaq files generated from the sequencing were detected using FASTQC software (v0.11.7). The software cutadapt (v1.18) was used to remove the Illumina adaptor sequence from the reads, the reads with a quality value lower than 20 and reads with sequence length lower than 25. The trimmed sequences were aligned with the mouse genome GRCm38 (GENCODE database) using STAR alignment software (v2.7.1a) (30), following the default parameters. The bam files generated from the alignment were sorted with SAMtools (v1.9) and subsequently used for the analysis of Differentially Expressed Genes (DEGs). DESeq2 package with the featureCounts program (31) in R (v3.5.1) was used for the identification of DEGs. To improve the results *apeglm* R package was used (32). The DEGs list was screened with padj< 0.05, |log_2_| fold change ratio > 1. To avoid false positive results the mean gene count among replicates of the same condition was set at ≥ 100 and the genes without annotation were removed. The Differentially Spliced Variants (DSVs) were detected and calculated using rMATS (v4.0.2) software (33) following the default parameters. The DSVs data were further processed with Excel (version 2016). The lists of DSVs were screened with an FDR< 0.01 and |∆Ψ| ≥ 0.1. To avoid false positive results, the mean of the included and skipped junction counts (IJC and SJC) for conditions was set at ≥ 20, which defines the threshold count. Gene enrichment analysis was performed using Enrichr browser (34). For the detection of the splice site strength the MaxEntScore browser was used comparing the splice sites adjacent to the skipped exons (35). For the research of enriched motifs STREME software was used (36). For the detection of intron retention iREAD was used (37).

### FLAG-CD2BP2 immunoprecipitation assay and liquid chromatography-mass spectrometry

For the FLAG-CD2BP2 immunoprecipitation assay the HEK293T CD2BP2^-/-^ cells were transfected with pcDNA3.1 vector overexpressing human CD2BP2 protein with a FLAG peptide at the C-terminal end. The transfected cells were lysed with 20 mM HEPES pH 7.5, 150 mM KCl, 10 mM MgCl_2_, 0.5 mM EGTA, 1% v/v NP-40 (Igepal), 1 mM DTT (freshly added), 1 tablet/10 ml Proteinase Inhibitor and Murine RNase inhibitor (NEB). At least 2 mg of lysate protein were added to 60 µl of anti-FLAG slurry beads (Anti-DYKDDDDK Tag (L5) Affinity Gel Antibody, BioLegend) previously washed with pulldown washing buffer (20 mM HEPES pH 7.5, 150 mM KCl). The suspension was incubated for 3–5 hours under agitation at 4°C. After three washing steps, performed with the washing buffer, the interacting proteins were eluted using 50 µl of elution buffer, 20 mM HEPES pH 7.5, 150 mM KCl and 1.5 µg/µl 3XFLAG peptide (BACHEM), for 30 min at 4°C. The eluate was supplemented with SDS gel-loading buffer and boiled at 95°C for 5 minutes, then the protein were separated using pre-casted gradient SDS-PAGE gel (4–20%, Bio-Rad). 26 bands were cut from the gel and tryptic in-gel digestion was performed. Dried peptides were reconstituted in 10 μl 0.1% TFA, 5% acetonitrile in water and 6 µl were analyzed by a reverse-phase capillary nano liquid chromatography system (Ultimate 3000) connected to an Orbitrap Velos mass spectrometer (Thermo Fisher Scientific). LC separations were performed on a capillary column (Acclaim PepMap100 C18, 2 μm, 100 Å, 75 μm i.d. × 25 cm; Thermo Fisher Scientific) at an eluent flow rate of 300 nl/min. Mobile phase A contained 0.1 % formic acid in water, and mobile phase B contained 0.1% formic acid in 80 % acetonitrile/20 % water. The column was pre-equilibrated with 5 % mobile phase B followed by an increase of 5–44 % mobile phase B over 40 min. Mass spectra were acquired in a data-dependent mode using a single MS survey scan (m/z 350–1500) with a resolution of 60,000 in the Orbitrap, and MS/MS scans of the 20 most intense precursor ions in the linear trap quadrupole with a normalized collision energy of 35. The dynamic exclusion time was set to 60 s and automatic gain control was set to 1x10^6^ and 5.000 for Orbitrap-MS and LTQ-MS/MS scans, respectively. The database search against the human subset of the SwissProt protein database was done using the software Mascot Distiller (Matrix Science). A maximum of two missed cleavages was allowed and the mass tolerance of precursor and sequence ions was set to 10 ppm and 0.35 Da, respectively. Methionine oxidation, acetylation (protein N-terminus) and propionamide (C) were used as variable modifications. A significance threshold of 0.05 was used based on decoy database searches.

### Immunoprecipitation assay, on-bead crosslinking and crosslinking analysis

The immunoprecipitation for the crosslinking experiment was performed as previously described but in a larger scale: 10^7^-10^8^ transfected cells and a ~ 100 mg of protein lysate. The crosslinking reaction on-beads was performed following the protocol used in previous publication (38).

The crosslinked peptides were pre-fractionated by a strong cation exchange (PolySULFOETHYL A™ column, PolyLC INC.) or by size exclusion chromatography (Superdex™ 30 Increase 3.2/300 column, GE Healthcare) on an Agilent 1260 Infinity system. LC-MS analysis was performed using an UltiMate 3000 RSLC nano LC system coupled on-line to an Orbitrap Fusion Lumos mass spectrometer (Thermo Fisher Scientific). Reversed-phase separation was performed using a 50 cm analytical column (in-house packed with Poroshell 120 EC-C18, 2.7µm, Agilent Technologies) with 80–120 min gradient. Crosslink acquisition was performed using a LC-MS2-MS3 method. The following parameters were applied: MS resolution 120,000; MS2 resolution 60,000; charge state 4–8 enable for MS2. Data analysis was performed using XlinkX standalone (39) with the following parameters: minimum peptide length = 6; maximal peptide length = 35; missed cleavages = 3; fix modification: Cys carbamidomethyl = 57.021 Da; variable modification: Met oxidation = 15.995 Da; DSSO crosslinker = 158.0038 Da (short arm = 54.0106 Da, long arm = 85.9824 Da); precursor mass tolerance = 10 ppm; fragment mass tolerance = 20 ppm. MS2/MS3 spectra were searched against a reduced UniProt human database derived from proteins identified by a standard proteomic measurement (containing 656 protein sequences). Results were reported at 1 % FDR at crosslinked spectrum match (CSM) level. The threshold for the n-score of MS2 and MS3 was set to <10^-10^ and <10^-13^ for peptide a and b, respectively.

## Results

### CD4^+^ T cell specific deletion of CD2BP2/U5–52K compromises T cell development

**Figure 1:**
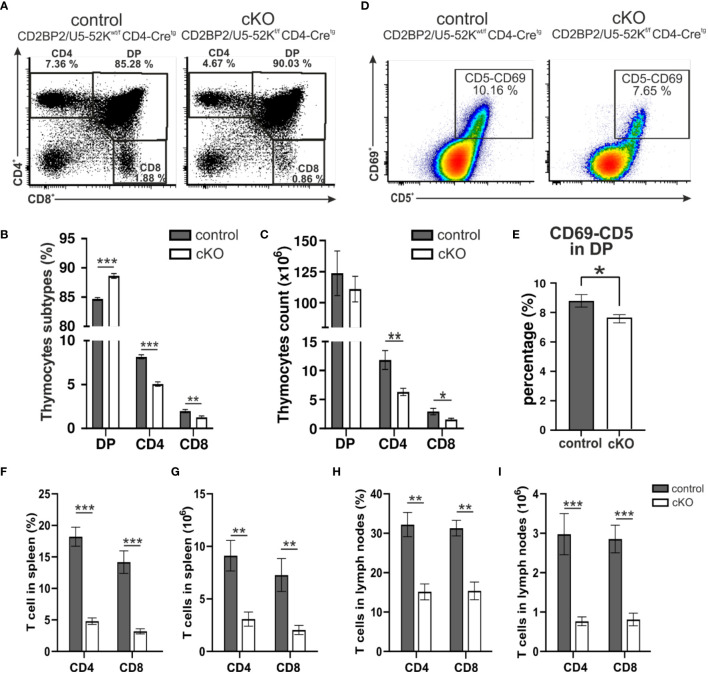
Analysis of T cells. **(A)** A representative flow cytometric dot plot of T cells from the thymus of a control mouse (CD2BP2/U5-52K^wt/f^ CD4-Cre^tg^) and a conditional knockout (cKO) mouse (CD2BP2/U5-52K^f/f^ CD4-Cre^tg^) is shown. The representation shows percentages of three subpopulations: single positive CD4^+^ (CD4), single positive CD8^+^ (CD8), and double positive (DP). **(B)** Bar diagram representing the percentage of CD4, CD8 and DP subpopulations in thymus of control (n = 6) and cKO (n = 8) mice. **(C)** Bar diagram representing the cell count of the thymocytes subpopulation for control (n = 5) and cKO (n = 6). **(D)** A representative flow cytometric dot plot of quadruple positive T-cell subpopulation CD4^+^, CD8^+^, CD5^+^ and CD69^+^ is shown. **(E)** Bar diagram representing the percentage of quadruple positive T-cells subpopulation in thymus of control (n = 6) and cKO (n = 8) mice. **(F, H)** Bar diagrams representing the percentage of the CD3^+^-CD4^+^ and CD3^+^-CD8^+^ populations in spleen **(F)** and peripheral lymph nodes. **(G–I)** Bar diagrams representing the CD3^+^-CD4^+^ and CD3^+^-CD8^+^ populations cell counts calculated in spleen **(G)** and lymph nodes. All the bar diagrams represent the mean of values detected in control (n = 6) and cKO (n = 8) mice. The error bars represent the standard error (SEM). Statistical analysis was performed using GraphPad Prism, using non-parametric, unpaired Mann-Whitney test. p value description: *p ≤ 0.05; **p ≤ 0.01; ***p ≤ 0.001; no stars: not significant.

Given CD2BP2/U5–52K´s role in embryonic development and in maintaining cellular integrity in the kidney (26), we asked whether CD2BP2/U5–52K would compromise the differentiation and functional integrity of T cells, a cell type that employs a stringent differentition program after birth. We crossed CD2BP2/U5–52K^flox/flox^ mice, described in our previous publication (26), with CD4-Cre mice (28). To validate the lack of CD2BP2/U5–52K in CD2BP2/U5–52K^f/f^ CD4-Cre^tg^ mice, referred as conditional knockout (cKO), we performed a RNA quantitative analysis via RT-qPCR on different subpopulations from thymus and peripheral organs (spleen and lymph nodes). Thymic double negative CD4^–^-CD8^-^ (DN), double positive CD4^+^-CD8^+^ (DP), single positive CD4^+^ and CD8^+^ cells as well as peripheral CD4^+^ T cells were isolated (**Supplementary Figure 1A**). The data shows that *CD2BP2/U5–52K* transcript is barely expressed in single positive (SP) T cells of CD2BP2/U5–52K^f/f^ CD4-Cre^tg^ mice in both thymus and peripheral organs (**Supplementary Figure 1A**). Thymi from cKO (CD2BP2/U5–52K^f/f^ CD4-Cre^tg^) and control (CD2BP2/U5–52K^wt/f^ CD4-Cre^tg^) mice do not show apparent differences in neither size/morphology (**Supplementary Figure 1B**) nor in the total cell count (control: 172.42 ± 21.29 x 10^6^ and cKO: 152.82 ± 13.21 x 10^6^, P > 0.05). However, the data of both single positive (SP) T cell subsets of cKO, CD4^+^ and CD8^+^, reveals a drastic reduction in the number and percentage of cells (**Figures 1A–C**; **Supplementary Figures 1C, D**), potentially arising from a dysfunction in differentiation, proliferation and/or apoptosis. We then assessed the expression of the activation markers CD69^+^ and CD5^+^ in DP cells, knowing that both markers are up-regulated during positive selection, a period where presentation of self-MHC-peptide to the αβTCR occurs. The analysis of the quadruple positive population (CD69^+^, CD5^+^, CD4^+^, CD8^+^), as represented in **Figures 1D, E**, shows a mild but significant reduction of T cells in the cKO mice in comparison to control mice. Additional comparisons with mice not expressing Cre (CD4-Cre^-^) validated our results since they show a comparable phenotype to the control group (**Supplementary Figures 1C, D**).

In conclusion, thymocytes in mice lacking CD2BP2/U5–52K display a strong reduction in T cell count and frequency of CD4^+^ and CD8^+^ thymocytes. The ratio between CD4^+^ and CD8^+^ is not altered suggesting that CD2BP2/U5–52K ablation affects both SP populations equally. Furthermore, the mild reduction of CD69 expression in cKO mice suggests a potential alteration in the positive selection capability of naïve T cells in the absence of CD2BP2/U5–52K.

**Figure 2:**
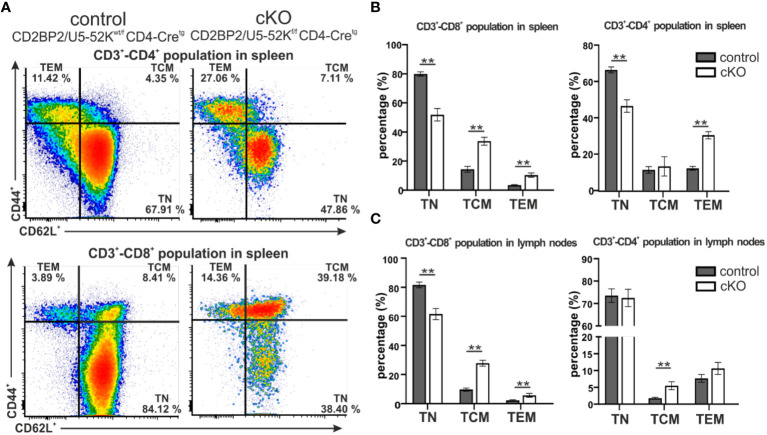
Analysis of naïve and memory T cells composition in spleen and peripheral lymph nodes. **(A)** Representative flow cytometric dot plots of T cells from spleen of control (CD2BP2/U5-52K^wt/f^ CD4-Cre^tg^) and cKO (CD2BP2/U5-52K^f/f^ CD4-Cre^tg^) mice. For the subtype selection CD62L and CD44 receptors in CD3^+^-CD4^+^ and CD3^+^-CD8^+^ populations were detected. The subtypes detected are naїve T cells (TN, CD62L^+^-CD44^-^), central memory T cells (TCM, CD62L^+^-CD44^+^), effector memory T cells (TEM, CD62L^-^-CD44^+^). **(B, C)** Bar diagrams representing the percentage of TN, TCM and TEM subtypes in control and cKO mice, in spleen **(B)** and lymph nodes **(C)**. All the bar diagrams represent the mean of values detected in control (n = 5-6) and cKO (n = 5-6) mice. The error bars represent the standard error (SEM). Statistical analysis was performed using GraphPad Prism, using non-parametric, unpaired Mann-Whitney test p value description: *p ≤ 0.05; **p ≤ 0.01; ***p ≤ 0.001; no stars: not significant.

**Figure 3:**
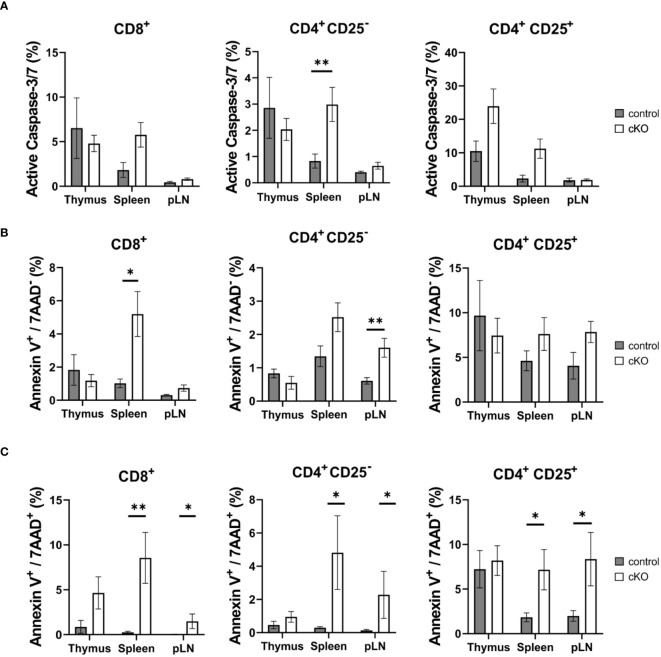
T cell apoptosis assay for CD8^+^, Tconv (CD4^+^-CD25^-^) and CD4^+^ Treg (CD4^+^-CD25^+^) derived from thymus, spleen and peripheral lymph nodes (pLN). From control mouse (CD2BP2/U5-52K^wt/f^ CD4-Cre^tg^, gray) and conditional KO (cKO, white) mouse (CD2BP2/U5-52K^f/f^ CD4-Cre^tg^) it was analyzed the caspase activity **(A)**, the early **(B)** and late **(C)** apoptosis. All the bar diagrams represent the mean of values detected in control (n = 5-6) and cKO (n = 7-8) mice. The error bars represent the standard error (SEM). Statistical analysis was performed using GraphPad Prism, using Mann-Whitney test. p value description: *p ≤ 0.05; **p ≤ 0.01; ***p ≤ 0.001; no stars: not significant.

Subsequently, we investigated the consequences for mature T cells in the peripheral organs, spleen and lymph nodes. T and B cells from these organs were discriminated via staining of the surface markers CD3 and B220. The gating strategy is depicted in **Supplementary Figure 1E** (upper panel). Similar to what we observed in the number of matured cells in the thymus, both CD4^+^ and CD8^+^, are strongly reduced in the homozygous mice, when either comparing them to CD2BP2/U5–52K^wt/f^ CD4-Cre^tg^ (**Figures 1F–I**) or CD4-Cre^-^ control mice (**Supplementary Figures 2A–F**). In contrast, B cell numbers are not significantly changed in both spleen and lymph nodes. Consequently, the T/B cell ratio in peripheral lymphoid organs is considerably reduced. In conclusion, our data indicates that CD2BP2/U5–52K deletion at the early stage of DP development compromise the transition of this population to SP cells in the thymus, and that the reduction in CD4^+^ or CD8^+^ T cell numbers is exacerbated in the peripheral lymphoid organs. Considering that the lack of CD2BP2/U5–52K induces T cell lymphopenia in both spleen and lymph nodes (**Figures 1B, C, F–I**), we wondered whether the ratio between naïve and memory T cells in both CD4^+^ and CD8^+^ populations is affected in the absence of CD2BP2/U5–52K. Differences between naïve and memory T cells in cKO and control mice are shown as exemplary flow cytometric dot blots in **Figure 2A**; **Supplementary Figure 3A**, with a quantitative analysis of their relative abundancies shown in **Figure 3B** for T cells of the spleen and in **Figure 3C** for lymph node populations. The CD3^+^-CD8^+^ population lacking CD2BP2/U5–52K (cKO) shows a significant increase of both central memory T cells (TCM) and effector memory T cells (TEM) in comparison to control mice. Note as well the significant reduction of naïve T cells (TN) in cKO mice (**Supplementary Figures 3B, C** left). For CD3^+^-CD4^+^ lymphocytes the effect is less severe, with a significant increase of TEM observed in the spleen while in the lymph nodes, only TCM are significantly increased. Furthermore, a significant reduction of TN in the CD3^+^-CD4^+^ population is only detected in the spleen (**Supplementary Figures 3B, C** right).

In conclusion, we could show that CD2BP2/U5–52K function influences differentiation and maturation of T cells in the thymus, reflected by lower relative and total numbers of T cells. The reduction of mature T cells is more prominent in the peripheral organs as the cKO/control ratio value of 0.53 for SP T cells in the thymus is reduced to 0.23 and 0.34 in spleen and peripheral lymph nodes, respectively. Moreover, mice lacking CD2BP2/U5–52K show an altered naïve/memory T cell compartment. In fact, both CD4^+^ and CD8^+^ populations in spleen and lymph nodes contain increased levels of of memory T cells in the cKO compared to the control, indicating a potential compensatory effect due to T cell lymphopenia (40).

### CD2BP2/U5–52K depletion in T cells increases apoptosis and blocks proliferation

**Figure 4:**
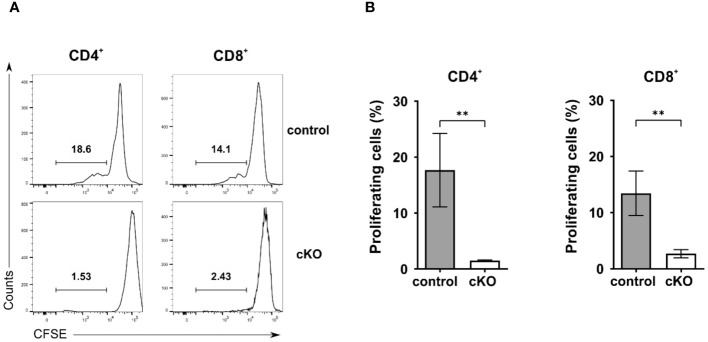
Proliferation analysis of T cells. A-B) Proliferation analysis of CD4^+^ and CD8^+^ T cells from control and conditional KO (cKO) mice. **(A)** Representative histograms and **(B)** summary bar graphs of CFSE-labeled CD4 and CD8 cells stimulated with anti-CD3 for 72 h. Bar diagrams represent the mean of values detected in cells from control (n = 5) and cKO (n = 5) mice. The error bars represent the standard error of mean (SEM). Mann-Whitney test was performed for statistical analysis using GraphPad Prism. p value description: **p ≤ 0.01.

Given the reduced number of T cells in the periphery, we asked the question whether T cells from homozygous cKO mice are more prone to apoptosis than those from control mice. We isolated CD8^+^, Tconv (CD4^+^-CD25^-^), and Treg cells (CD4^+^-CD25^+^) from thymus, spleen and peripheral lymph nodes from both control and cKO mice. We analyzed caspase 3/7 activity and Annexin V and 7AAD for the detection of early and late apoptotic events, respectively **Figures 3A–C**; **Supplementary Figure 4**). The detection of caspase activity and Annexin V (**Figures 3A, B**) indicates that the cKO T cells derived from the peripheral organs (spleen and lymph nodes) show increased apoptotic phenotypes in comparison to the control cells. This effect became even more evident when analyzing 7AAD staining, which indicates membrane rupture **Figure 3C**). For SP cells from the thymus we did not see significant differences between control and cKO cells, indicating that CD2BP2/U5–52K protein may still be available at this stage **Figure 3A**). Furthermore, we monitored cell growth of peripheral T cells by stimulating T cells *in vitro* with anti-CD3 antibodies and monitoring proliferation by CFSE staining. Strongly reduced proliferation of both, CD4^+^ and CD8^+^ T cells isolated from cKO mice was detected compared to the respective T cells from control mice (**Figures 4A, B**).

### CD2BP2/U5–52K-deficient cells increase the expression of genes related to the p53 pathway

**Figure 5:**
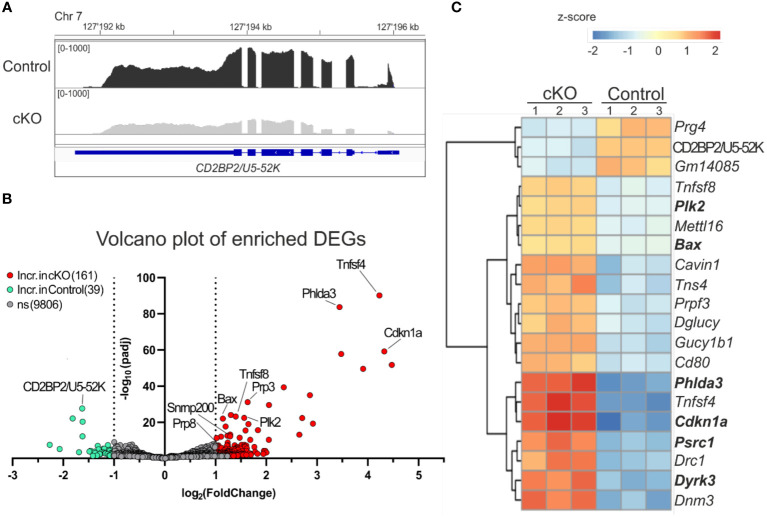
Analysis of differentially expressed genes. **(A)** IGV representation of the read counts sum of *CD2BP2/U5-52K* gene in gray for cKO (CD2BP2/U5-52K^f/f^ CD4-Cre^tg^) and in black for Control (CD2BP2/U5-52K^wt/f^ CD4-Cre^tg^ and CD2BP2/U5-52K^f/f^ CD4-Cre^-^) mice samples. The read count of the three samples per conditions were merged using Samtools software. The CD2BP2/*U5-52K* gene structure is schematically represented in blue, the larger lines represent the exons. **(B)** Volcano plot representation of the Differential Expression Genes (DEGs) detected in the RNA-seq analysis. The significant genes are increased in the Control samples (green) or in the cKO samples (red), which has a padj value < 0.05 and a log2(foldchange) >± 1|. **(C)** Heat map of the 20 most significant DEGs, based on the p-value adjusted, detected between the two conditions. Analysis and visual representation performed with DESeq2 software in R.

It has been previously shown that the lack of CD2BP2/U5–52K in mice leads to embryonic lethality during the period between E9.5-E11.5, coinciding with changes in RNA expression and splicing (26). To better understand the functional consequences of CD2BP2/U5–52K ablation for gene expression signatures, we performed RNA-sequencing analysis of CD4^+^ T cells derived from spleen and peripheral lymph-nodes of cKO and control mice (described in Material and methods section). The analysis of the reads in coding exons of *CD2BP2/U5–52K* across both conditions confirms the low mRNA level of *CD2BP2/U5–52K* in the cKO mice sample in comparison to the control (**Figure 5A**). Moreover, *CD2BP2/U5–52K* expression is confirmed for the control condition (**Supplementary Figure 5A**-left) and was further validated by RT-qPCR experiments (**Supplementary Figure 5A**-right). Moreover, principal component analysis (PCA) indicates similarity among replicates of the same conditions (**Supplementary Figure 5B**). Therefore, we proceeded with the analysis of Differentially Expressed Genes (DEGs) between paired conditions, which identified 200 significant DEGs from a total of 10006 genes. Of those 200 DEGs, 39 (19.5%) are increased in the control mice, including *CD2BP2/U5–52K*, while the vast majority, 161, (80.5%) are increased in the cKO mice (**Figure 5B**; **Supplementary Figure 5C** and **Supplementary Table 1**). To analyze if the genes with increased expression in the cKO mice are related to specific cellular pathway we used Enrichr program (34), which identified a significant enrichment of genes related to p53 pathway/activity (using BioPlanet, KEGG and Panther database, **Supplementary Figure 5D**). For a closer inspection we selected the 20 most significant DEGs, based on adjusted p-value (**Figure 5C**). Of those the most prominent proapoptotic genes identified are *Bax* (Apoptosis regulator BAX) and *Cdkn1a* (cyclin-dependent kinase inhibitor 1a). BAX protein is part of the BCL-2 family and is known to trigger apoptosis via permeabilization of the mitochondrial outer membrane after p53 activation (41), while the gene *Cdkn1a* encoding for the protein p21 (Waf1, Cip1) is regulated by p53 to induce cell cycle arrest (42, 43). Moreover, in the same list less well characterized proteins with tumor suppressor activity and involved in the p53 pathway were identified such as the *Phlda3* (pleckstrin homology like domain, family A, member 3) (44) and *Plk2* (Polo like kinase 2) (42). Additional apoptotic genes with significant increases in gene expression in the cKO were identified as *Gadd45g*, *Tnfrsf10b*, *Pmaip1* and *Zmat3*, encoding for the proteins GADD45G (growth arrest and DNA damage-inducible protein GADD45 gamma), DR5 (Death Receptor 5), NOXA (Phorbol-12-myristate-13-acetate-induced protein 1) and PAG608/WIG1 (Zinc finger matrin-type protein 3), respectively (45–48).

Interesting, we detected two cytokine genes *Tnfsf4* and *Tnfsf8* (Tumor necrosis factor ligand superfamily members 4 and 8, respectively) in the list of the 20 most significant DEGs. Both proteins are implicated in memory T cell development (49, 50), which is in agreement with the increased amount of these cell types found in the cKO mice (**Figures 2B, C**). In conclusion, our results indicate that CD2BP2/U5–52K KO T cells, once they have reached the peripheral lymphoid organs, have acquired a state where they can compensate for the splicing defects in the absence of CD2BP2/U5–52K to a certain extent. However, the observed increase in apoptosis and reduction in proliferation raises the question of whether splicing defects can account for the observed phenotype.

### CD2BP2/U5–52K regulates the skipping of exons in many apoptosis-relevant genes

**Figure 6:**
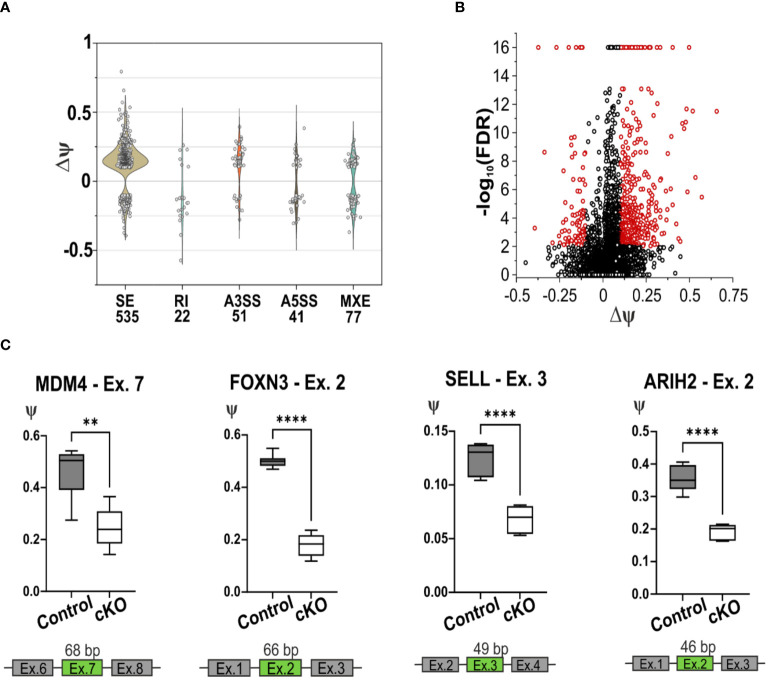
Splicing deficiency in cKO T cells. **(A)** Violin plot of the splicing events detected using rMATS software in the CD4^+^ T cells of cKO and Control mice. The x-axis represents the type and the quantity of significative splicing events, named Differential Splicing Events (DSVs), while the y-axis represents the difference of the percentage splice-in (∆ψ) detected in the two conditions, (ψControl – ψcKO). The splicing events are defined as Skipping Exon (SE), Retain Intron (RI), Alternative 3’ Splice Site (A3SS), Alternative 5’ Splice Site (A5SS), Mutually Exclusive Exons (MXE). **(B)** Volcano plot representation of the detected SE, in red are highlighted the significant events, FDR < 0.01 and |∆ψ| ≥ 0.1. **(C)** Graphic representation of the validated skipping exon events of the gene Mdm4, *Foxn3*, *Sell* and *Arih2*. In green are highlighted the exons that are alternatively spliced in cKO mice. Statistical analysis was performed using GraphPad Prism, using non-parametric, unpaired Mann-Whitney test. p value description: *p ≤ 0.05; **p ≤ 0.01; ***p ≤ 0.001; ****p≤ 0.0001, no stars: not significant.

Given the primary role of CD2BP2/U5–52K as a splicing factor we decided to analyze the Differential Splicing Variants (DSVs) affected by the lack of CD2BP2/U5–52K. Our approach [using rMATS (33)] identified several splicing events including skipped exon (SE), retained intron (RI), alternative 3’ or 5’ splice sites (A3SS ad A5SS) and mutually exclusive exons (MXE). We used a count threshold to verify the quality of the data as described in the Materials section. From a total of 55372 splicing events identified from a total of 8192 genes, 726 DSVs were considered significantly different for the two conditions. Thus 1.3 % of the total splicing events detected in CD4^+^ T cells are influenced by CD2BP2/U5–52K function. Of those, the SE events are the most abundant with 535 significant hits, 73.7 % of all the DSVs detected (**Supplementary Figure 6A**; **Supplementary Table 2**). From all the observed SE events, the difference of the percentage spliced-in variants (∆ψ) represents the difference between the two conditions (control versus cKO). The majority of hits have a positive value (444 out of 535, ~ 83 %) indicating that for CD4^+^ T cells the lack of CD2BP2/U5–52K leads to an increased number of skipped exons (**Figures 6A, B**). Considering the low number of differential splicing events in the RI, A3SS, A5SS and MXE categories (**Supplementary Figure 6B**), an in-depth analysis was performed uniquely for the SE data. In this case, to seek whether CD2BP2/U5–52K exerts specificity at the transcript level we analyzed the data with regard to recurrent sequence motifs, splice site strength, intron retention and intron-exon length. By using STREME (36) analysis of the alternatively spliced exon sequences, a single motif of the sequence 5’-CCGTTA-3’could be defined as slightly enriched for the SE hits in comparison to the whole set of SE (p-value of 0.013, **Supplementary Figure 6C**). The enrichment percentage for this sequence motif is 3.8 %, indicating that the large majority of SE events seen in cKO cells is not caused by RNA sequence recognition. Similarly, no relevant difference was detected for the strength of the 3’ and 5’ splice sites adjacent to the skipped exon [using MaxEntScore (35)], or in intron retention [using iREAD (37)]. Finally, we analyzed the length of the skipped exons and adjacent introns comparing the CD2BP2/U5–52K enriched SE with all the detected SE. There is a significant increased length of up- and downstream introns (**Supplementary Figure 6D**). This data points towards a structural or kinetic aspect of exon skipping in the absence of CD2BP2/U5–52K. No influence of exon length was observed.

To independently confirm the most relevant hits from the RNA-seq derived data, RT-qPCR was performed, validating 7 out of 10 DSVs in the SE list (**Figure 6C**; **Supplementary Figures 6E–G**). This validation was obtained using RNA from CD4^+^ T cells from spleen and lymph nodes of cKO and Control mice. In all the selected SE events, the lack of CD2BP2/U5–52K induces skipping of the corresponding exons. One of the most relevant splicing variants detected by RNA-seq and validated by RT-qPCR is the skipping of exon 7 in the *Mdm4* gene, which encodes for a protein involved in regulating the p53 pathway. Two isoforms of this protein exist, the long MDM4-FL and the short and unstable MDM4-S isoform, which lacks exon 7 and generates a premature stop codon. Between the two, only MDM4-FL is an efficient inhibitor of the apoptotic activity of the tumor suppressor protein p53 (51, 52). The RT-qPCR data validate the diminished presence of exon 7 in CD2BP2/U5–52K depleted T cells (**Figure 6C**), indicating a potentially increased protein expression of the MDM4-S isoform, as it was shown previously that mRNA and protein levels of MDM4 correlate (53). We could further confirm the skipping of exon 2 in the gene *Foxn3* upon loss of CD2BP2/U5–52K (**Figure 6C**). The gene encodes for the protein Forkhead box protein N3, which acts as a transcription repression and potent tumor suppressor (54). We also validated the skipping of exon 3 in *Sell* transcript, which encodes for CD62L or L-selectin. The alternative splicing of the 49 bp in the exon 3 generates two isoforms with different C-terminus, known as L-selectin-c and L-selectin-v1. The transcripts were shown to differ in the speed of T cell rolling and shedding upon PMA induction (55). Both transcripts are detected by the MEL14 antibody clone (55), indicating that interference with detection of CD62L in the naïve/memory T cells experiment should not be expected. Furthermore, the exon 2 of the gene *Arih2* was also differentially spliced, which encodes for tumor suppressor TRIAD1 protein (56).

Many others DSVs, depicted in the **Supplementary Figure 6E**, were validated as for example the exons 4, 5 and 6 in the *Ptprc* transcript, encoding for CD45. Exons 4, 5 and 6 are variable cassette exons, and are known to be alternatively spliced in both human and mouse, which gives rise to 5 distinct isoforms involved in T cell signaling and development (57). The CD45 transcript without those exons encodes for the isoform CD45RO which is abundantly expressed in memory T cells (58), in line with the observations of increased amounts of memory T cells in the absence of CD2BP2/U5–52K (**Figure 2**). The splicing of *Sp100* is also affected by CD2BP2/U5–52K and we were able to validate the reduced level of exon 11 inclusion for this transcript. *Sp100* is implicated in transcriptional regulation of chromatin. Several *Sp100* human isoforms have been characterized, which mostly differ in their C-termini (59), while no mouse *Sp100* isoforms were reported so far. Finally, alternative splicing validation was performed on exon 6 of the *Stk19* transcript. The Stk19 protein was discovered to regulate NRAS activity, promoting oncogenic NRAS-mediated melanocyte malignant transformation (60).

### The role of CD2BP2 as a spliceosomal assembly factor

**Figure 7:**
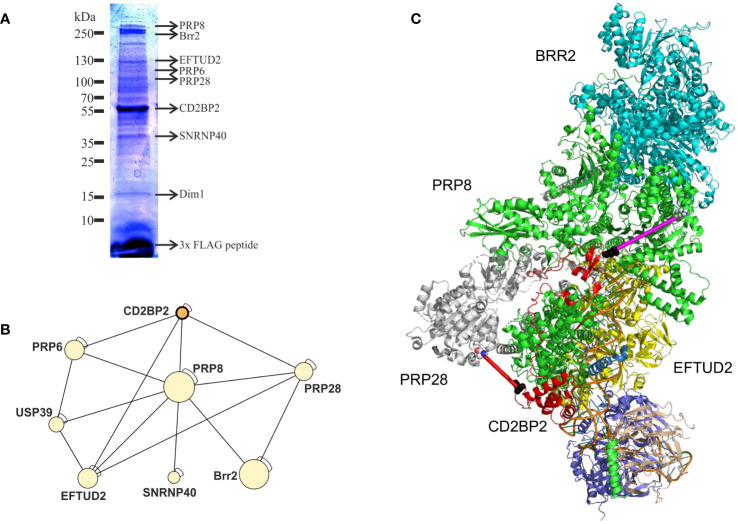
CD2BP2 is part of the U5 snRNP. **(A)**, Immunoprecipitation (IP) experiment. The proteins enriched in an IP against FLAG-tagged-U5–52K were separated using SDS-PAGE and after tryptic in-gel digestion proteins from individual bands were identified by mass spectrometry. Selected proteins that are part of the 20S U5 snRNP and the gel band in which they were identified are highlighted. **(B)** Graphic representation of proteins forming the crosslinked U5–52K complex (CCC). **(C)** Structure of the U5 snRNP with CD2BP2 shown in red. In order to be able to mark the cross-links of K188 of CD2BP2 to K385 of PRP28 (red line, 25.1 Å) we had to replace the only partially resolved structure of CD2BP2 in the experimental structure (8Q7X) by a contiguous fragment 64–200 from CD2BP2 obtained from AlphaFold 3 predictions of the CD2BP2/PRP8 complex. PRP8 is shown in green and the distance between D64 of CD2BP2 and K892 of the RT domain of PRP8 was determined to be 41.8 Å (shown as a magenta line). K892 displays a cross-link to K26, which is part of the intrinsically disordered domain of CD2BP2. However, this crosslink between K26 (CD2BP2) and K892 (PRP8) indicates that the N-terminus of CD2BP2 is oriented towards the RT domain of PRP8 and the helicase BRR2, thereby localizing proximal to one of the TSSC4 docking sites of PRP8.

With the aim to confirm the role of CD2BP2/U5–52K in the spliceosome and to identify interaction partners we performed affinity-purification with and without subsequent crosslinking and then analyzed samples by mass spectrometry. We first deleted the CD2BP2/*U5–52K* gene in HEK cells via CRISPR/Cas9 (**Supplementary Figure 7A**). Subsequently, FLAG-tagged-U5–52K protein was transiently overexpressed in these cells. An immunoprecipitation assay (IP) using anti-FLAG resin was then used for enrichment of CD2BP2/U5–52K and potential binding partners. Proteins were eluted by an excess of FLAG-peptide and separated by SDS-PAGE (**Figure 7A**). The whole gel lane was excised into 26 bands and after tryptic in-gel digestion proteins were identified by liquid chromatography-mass spectrometry (LC-MS). Ranking all identified proteins detected in three IPs by their relative intensities using the corresponding iBAQ values (61) shows once again that CD2BP2/U5–52K captures proteins of the U5 complex with high abundancy (**Supplementary Figure 7B**). Those data confirm previous results that assign the protein to this particular snRNP (62). To confirm that direct contacts of U5–52K comprise mostly proteins of the U5 snRNP and in order to gain structural insights on the core CD2BP2/U5–52K complex, we performed an anti-FLAG IP under conditions of on-bead crosslinking by DSSO. After tryptic digest, the crosslinked peptides were enriched via ion exchange chromatography and analyzed using mass spectrometry. In total, four experiments were performed: two technical (TR1 and TR2) and two biological replicates (BR1 and BR2) (**Supplementary Table 3**). The visualization tool xiNET (63) allows the identification of the complex of proteins directly or indirectly crosslinked via CD2BP2/U5–52K (**Supplementary Figure 7C**). The complex of crosslinked proteins interacting with CD2BP2/U5–52K, identified across all technical and biological replicates were considered as part of the crosslinked CD2BP2/U5–52K complex (CCC) (highlighted in dark gray in **Table 1**) and a schematic representation is depicted in **Figure 7B**. Most of these proteins in the CCC are described as part of the 20S-U5 particle confirming that CD2BP2/U5–52K is part of this particular snRNP. Given the absence of almost all U4/U6 snRNP or tri-snRNP specific factors, with the exception of USP39, we conclude that CD2BP2/U5–52K dissociates from U5 upon stable tri-snRNP formation, as previously proposed (62). Interestingly, the recently discovered U5 snRNP assembly factor TSSC4 (64, 65), was identified in three out of four replicates.

**Table 1:** List of proteins found (x) in the crosslinked CD2BP2/U5-52K complex in technical (TR) and biological (BR) replicates.

	Protein name	TR1	TR2	BR1	BR2
20S U5 snRNP	U5-52K	x	x	x	x
	Brr2 - SNRNP200	x	x	x	x
	Prp28 - DDX23	x	x	x	x
	Prp6	x	x	x	x
	Prp8	x	x	x	x
	Snu114 - EFTUD2	x	x	x	x
	SNRNP40	x	x	x	x
	TSSC4	x		x	x
	Dim1 - TXNL4A				x
U5 snRNP assembly fact.	A2A2Q9 - AAR2		x		
U4/U6.U5 tri-snRNP	USP39 - SAD1	x	x	x	x
	SART1			x	x
B complex	IK - RED			x	x
Proteins involved in the spliceosome machinery	DHX38 - Prp16			x	x
	AQR				x
	ZRANB2				x
Proteins not associated with spliceosome machinery	BRIX1			x	x
	ACAD11			x	
	EAPP			x	
	BZW1				x
	SPEN	x			
	DYNC1I2			x	

The symbol x represents the identification of this protein in the crosslinked complex with CD2BP2/U5-52K.

To confirm a structural role of CD2BP2/U5–52K position in the U5 snRNP, we capitalized on the recently determined structures of the U5 snRNP (13, 14). We used an AlphaFold 3 (66) predicted structure of a complex between CD2BP2 and Prp8 and then replaced the CD2BP2 (partially) structured region (amino acids 64–230) in the U5 snRNP state IV structure (PDB: 8Q7X) (**Figure 7C**). Thereby, regions that are not resolved in the experimental structures, for example the loop 181–190, could be visualized, which helped to interpret the crosslinking data. Crosslinked peptides were considered relevant when identified in at least two out of the four replicates. Following those criteria, two regions of CD2BP2/U5–52K show crosslinking interactions with U5 snRNP proteins. The first region locates to the N-terminal part of CD2BP2/U5-52K, more precisely lysine 26, and shows interactions both with PRP6 (K585 within the HAT repeat 4) and PRP8 (K892 within the reverse transcriptase (RT)-like domain). This observation confirms previous studies that detected an interaction between PRP6 with the N-terminal region of CD2BP2/U5–52K (62). Furthermore, the placement of the N-terminus relatively close to the RT/Jab1 domain of PRP8 (**Figure 7C**, magenta line) anchors this part of CD2BP2/U5–52K close to the TSSC4 helix that docks onto PRP8, further suggesting that the two chaperones, CD2BP2/U5–52K and TSSC4 act in concert, at least during certain phases of assembly. The second region of CD2BP2/U5–52K that forms an intermolecular lysine crosslink is the loop 181–190 in the central part of CD2BP2/U5-52K, which crosslinked to PRP28 (K385, in proximity to the Q motif), EFTUD2 (K244, in the type-G domain) and PRP8 (K58). The crosslink to PRP28 is in agreement with the interface of the two molecules in the EM structure (**Figure 7C**, red line), while K244 of EFTUD2 is not compatible with the experimental complex. Again, the crosslink to PRP8 is very plausible, given that K58, which is at the very beginning of an unresolved stretch of PRP8, is succeeded by glutamate 59, which displays a Cα-Cα distance of 21.8 and 22.8. Å to K185 and K188, respectively. Interestingly, no relevant crosslinked peptides were identified for the C-terminal GYF domain of CD2BP2/U5–52K, which interacts with proline-rich peptides in SmB/B’ (67) and with Dim1 (19). This is in agreement with both reports on the U5 snRNP structure and is explained by the observation that placement of Dim1 as part of the tri-snRNP would be sterically hindered as long as the GYF domain of CD2BP2/U5–52K is bound.

## Discussion

### CD2BP2/U5–52K depletion alters splicing and transcription of genes involved in p53 signaling

CD2BP2/U5–52K impacts splicing and transcription of genes involved in the apoptotic response. We postulate that the cell death phenotype is caused by a splicing defect rather than an unanticipated role as a direct regulator of transcription or apoptosis. Transcript analysis shows that exon skipping is the main consequence of CD2BP2/U5–52K ablation, hinting at a limiting concentration of active spliceosomes. In line with this, neither specific RNA motifs nor differences in predicted splice site strength could be identified in the large set of skipped exons identified in the absence of CD2BP2/U5–52K. Several of the exon skipping events, some of which were confirmed via RT-qPCR, are linked to the p53 pathway. Of relevance, the alteration of exon 7 induces the formation of the MDM4-S (short) isoform, which in contrast to MDM4-FL (full-length isoform) is unable to inhibit p53 activity (52, 68) and leads to apoptosis. While we are missing the information at the protein level due to technical reasons, previous studies had shown that MDM4 mRNA and protein levels correlate during brain development (53). In this study, depletion of the pre-mRNA splicing regulator ARGLU1 resulted in upregulation of p53 by mis-splicing of MDM2 and MDM4. Along similar lines, other defects of the spliceosomal machinery, as the U4/U6.U5 tri-snRNP, or the absence of the splicing regulator PRMT5, have also been shown to influence the generation of MDM4-S (69, 70). Thus, MDM4 seems to qualify as a common target that senses splicing efficiency and once critical ratios of short-to-long isoform have been formed, commits cells to apoptosis. It is tempting to speculate that T cell apoptosis can be induced by MDM4-S isoform formation, indicating that the MDM4 splicing switch is functionally operative in immune cells. The absence of CD2BP2/U5–52K also alters the splicing of the *Arih2* and *Foxn3* genes, which encode for the tumor suppressor proteins TRIAD1 and CHES1, respectively (54, 56). TRIAD1 was shown to facilitate the degradation of MDM2, increasing the apoptotic activity of p53 (56), while CHES1 downregulates the expression of the oncogenic proteins PIM2 and E2F5 in cancer cells (71, 72). Little is known about the isoforms of Foxn3 and Arih2 but according to the Ensembl database transcripts containing exon 2 in either case may lead to nonsense mediated decay of the two proteins. Thus, in the absence of CD2BP2 when exon 2 is skipped, these two tumor suppressors are conceivably more active in promoting p53-mediated apoptosis of T cells.

### Critical role of CD2BP2/U5–52K in cellular homeostasis

CD2BP2/U5–52K deletion in T cells results in a strong lymphopenia that is caused by reduced proliferation and enhanced apoptosis of T cells. The reduced proliferation we observed *in vitro* is in line with the increased expression of the cyclin-dependent kinase inhibitor *Cdkn1a*/p21^Cip1/WAF1^, which is a broad regulator involved in cell senescence, apoptosis and cell cycle arrest (42, 43, 73). Aside from *Cdkn1a*, the depletion of CD2BP2/U5–52K upregulates the apoptotic genes *Bax*, *Plk2*, *Phlda3*, *Gadd45g*, *Tnfrsf10b*, *Pmaip1*. *Bax* and *Pmaip1* (enconding for NOXA) are pro-apoptotic family members of the Bcl-2 family that lead to mitochondrial outer membrane permeabilization, the characteristic event of the intrinsic apoptosis pathway (74), while *Tnfrsf10b* (DR5/TRAIL-R2) is a death domain-containing receptor for TNF-related apoptosis inducing ligand (TRAIL) (75). Thus, presumably both, intrinsic and extrinsic apoptosis pathways are activated by *CD2BP2/U5–52K* deficiency. Furthermore, inducement of PLK2 and PHLDA3 in cKO cells indicate that the mTOR/AKT pathway might be attenuated as a consequence of p53 activation (44, 76). In line with our results, alterations in another U5 snRNP protein, EFTUD2, influences apoptosis via p53, in this case through exon skipping and mis-splicing of the *Mdm2* gene (77). Mutations in EFTUD2 result in mandibulofacial dysostosis indicating an increased sensitivity of craniofacial development for altered splicing efficiency (78). Thus, we hypothesize that mutations in genes of the U5 snRNP or other components of the general splicing machinery are especially detrimental to cells that require splicing for rapid differentiation, as for example during embryonic development (79) or during cellular reprogramming upon immune receptor stimulation (this study). Still, depletion of CD2BP2/U5–52K in podocytes, a fully differentiated cell type required for renal function, compromises cellular integrity, albeit at a time scale of several month rather than weeks (26).

As a consequence of T cell death, a reduced T cell compartment often results in so-called homeostatic proliferation that is associated with the upregulation of activation and memory markers (80). Despite the reduction in cell proliferation, a significant increase in percentage of memory T cells was observed, which is in line with the observation that the memory T cell phenotype might be protected against apoptosis by overexpressing the anti-apoptotic Bcl members Bcl-x(L) and Bcl-2 (81). Moreover, our RNA-seq data indicates that the *Ptprc* transcript encoding CD45RO (57), as well as the *Tnfsf4* and *Tnfsf8* genes (49, 50), typical markers for memory T cells, are considerably upregulated in cKO animals when compared to the control, in agreement with previous results (82).

The question that has not been addressed in this study is whether the originally proposed interaction between CD2 and CD2BP2/U5–52K is of relevance *in vivo*. Certainly, the phenotypes between CD2 and CD2BP2/U5–52K KO mice are very different. Ablation of CD2 does not lead to T cell death or a proliferation deficit (83) as it is seen for CD2BP2/U5–52K knockout mice. Thymic development is not markedly altered and thymic as well as peripheral T cell numbers are unchanged in CD2 knockout mice, in contrast to what is observed in this study. Thus, the impact of CD2BP2/U5–52K on cellular function is much more profound than for CD2, in line with those studies that show that splicing regulation is of great importance for the differentiation, proliferation and migration of T cells (84). It is of course possible that CD2 fine-tunes T cell responses by engaging CD2BP2/U5–52K, for example by sequestering the nuclear GYF protein away from the nucleus to the membrane. However, previous studies with HeLa cells co-expressing CD2 and CD2BP2/U5–52K did not indicate a substantial co-localization of the two proteins, with CD2BP2/U5–52K predominantly localizing to the nucleus (21). Therefore, if CD2 and CD2BP2/U5–52K are to interact, additional signals, for example during certain phases of T cell development, possibly in an activation-dependent manner, would have to be invoked. Given that the interaction between the GYF domain of CD2BP2/U5–52K and the two proline-rich sequences in CD2 is of relatively low affinity (15, 85), CD2 seems to be unable to independently serve as a hub for CD2BP2/U5–52K. In contrast, in the nucleus the concentration of proline-rich sequences containing the PPG motif is high given the many proteins that contain this signature in their unstructured tails. Consequently, it has been shown that for example the core splicing protein SmB/B’ recruits CD2BP2/U5–52K and the WW domain containing protein FBP21 to its C-terminal tail in living cells. Thus, while an accessory role of CD2BP2/U5–52K in the function of CD2 is well possible its core function is independent of CD2 and relates to its role as a splicing factor. Its role as critical factor for U5 snRNP biogenesis and recycling has recently be corroborated by the isolation and structural characterization of several stages of the U5 snRNP, which shows how CD2BP2/U5–52K interacts with central components of the maturing spliceosome, most prominently PRP8 (13, 14). We have independently performed crosslinking mass spectrometry experiments with the U5 snRNP and could confirm the structurally described interactions (**Figure 7C**). Interestingly, we also find regions of CD2BP2/U5–52K that are part of intrinsically disordered segments of CD2BP2/U5–52K (K26, K185, K188) to crosslink to components of the U5 snRNP, supporting a prominent role of the protein as a chaperone and assembly factor of the spliceosome.

In summary, CD2BP2/U5–52K deficiency in T cells leads to a functionally relevant increase of exon skipping which potentially gives rise to alterations in gene expression profiles. This results in reduced proliferation, and enhanced T cell death by apoptosis explaining the T cell lymphopenic phenotype of the cKO mice (**Supplementary Figure 8**). The skewed T cell homeostasis points to a differential requirement for splicing for memory T cells compared to naїve and effector T cells that could be exploited in therapeutic settings. Constraining the growth of over-proliferative T cells in T cell leukemia by inhibition of the U2 snRNP components SF3B1 marked a proof-of-principle of this concept (86). Potentially, splicing inhibition targeting U5 snRNP components such as CD2BP2/U5–52K, could be exploited in similar ways to restrict T cell growth in leukemia (86) or autoimmunity (87).

## Data Availability

The datasets presented in this study can be found in online repositories. The names of the repository/repositories and accession number(s) can be found below: GSE208003 (GEO).
