# Supplementary material for: The nuclear GYF protein CD2BP2/U5–52K is required for T cell homeostasis
Source: Front Immunol. 2024 Sep 6;15:1415839. doi: 10.3389/fimmu.2024.1415839 (PMC11412891; doi:10.3389/fimmu.2024.1415839)
Supplement: Supplementary file 1 [file DataSheet1.pdf]

Supplementary material

Supplementary list 1

Suppl. list 1 List of primers paired used for genotyping detection and qPCR analysis

Primer pairs for qPCR	Forward sequence	Reverse sequence
Flox allele	GGCGTTGAGGTTTGAGTTGT	AACCCAGGAGTCATTTGCAC
CD4-Cre	CGATGCAACGAGTGATGAGG	GCATTGCTGTCACTT GGTCGT
CRISPR-Cas oligos 1	CATTGTCCTCCCACGTGACT	CCCGGGCTGTGTACAACTTA
CRISPR-Cas oligos 2	GCTTTCAGGACCTCAGATTCTTTAT	CAAGGATAGAGGAAAGCCAGATTAG
Sell – targeted ex.	CCTCTTCATTCCTGTAGCCGT	CAAAACTGCAGCAGACTGTGGG
Sell – control ex.	TTT CTC AAA GAT CAA AGA AGG TGA C	TGC CAG CCA AAT GAG AAA TGC
Bcl11b – control ex.	ATGCAAGCAGCCCTTCAACA	ATTCATGAGTGGGGACTGCG
Bcl11b – targeted ex.	CCTCCCACCTCCGTGATTACT	GTGCAAATGTAGCTGGAAGGC
Stk19 – targeted ex.	GAACGCTGGGGTCCTCAC	GGCCCAGGAGCTCTGACA
Stk19 – control ex.	CTG TGA TGG CCG ACC ATG TG	CGG GTC CCT GAA GCC ATA AG
Ptprc – targeted ex.	GACCCTATTTCTTAGGGGCACA	CATCACTGGGTGTAGGTGTTT
Ptprc – control ex.	CACTGAATCCACACCCCCAA	CACACCTGGTGGGTCAGTC
Foxn3 – control ex.	GACATTGATGCTGCCAGTGC	GGCTGCCGTCATCCCA
Foxn3 – targeted ex.	CCA GGA GTG ATA CAA AAT GGA GCG	TTA TGG CTG CCG TCA TCC CAA T
Vps37a – control ex.	TTGGAACATAGCTTGGAAGGC	AGCTTCGTGTGCAGCTACTTT
Vps37a – target ex.	TTGGAACATAGCTTGGAAGGC	TGAGTTCATGTTGCCTTTGCAT
Cln3 – target ex.	TGACATCCTCAAGCAGGAGC	GTGGAGATGGAGTTGCAGTCA
Cln3 – control ex.	GCC TTC ACT TGC TGC CTT AC	CAC ACA GGC TTA ACC CCA CT
Sp100 – control ex.	ACTCAGCAGTGGGGATTCTG	CTCCTCCTCTGTCTCTTCTCA
Sp100 – target ex.	ACTCAGCAGTGGGGATTCTG	TCCTGTCCTTTTCCGTCTTCTAA
Mdm4 – target ex.	CTGCTCAGACTCTCGCTCTC	ATGTCGTGAGGTAGGCAGTG
Mdm4 – control ex.	CGG TGC AAC AGA ATA CTC CA	TCC CAC TCC TCA AAA TCA AGG T
Arih2 – targeted ex.	GACCTATAAGGAGTCTGAGGGC	TGCCATAGGGAGGACGCA
Arih2 – control ex.	AGT TAA TTT CCA CTG GCA AGT CT	TTT GGA TGG ATT AGG TTG AAC TCG
U5-52K – targeted ex.	CCTGACCCTGGGTTCCTA	TTGCTGGACCCTTCCTCATC
Actin – control ex.	CCGCGAGCACAGCTTCT	GGGTACTTCAGGGTCAGGAT
Ube2d2a	AAGAGAATCCACAAGGAATTGAATG	CAACAGGACCTGCTGAACACTG

Supplementary list 2

Suppl. list 2 List of the gene and transcript IDs. Genes details refer to Ensembl release 102 (GRCm38).

Gene name	Gene ID	Transcript ID
Foxn3	ENSMUSG00000033713	ENSMUST00000222458
Mdm4	ENSMUSG00000054387	ENSMUST0000067429
Arih2	ENSMUSG00000064145	ENSMUST00000193552
Ptprc	ENSMUSG00000026395	ENSMUST00000183301
Sp100	ENSMUSG00000026222	ENSMUST00000153574
Stk19	ENSMUSG00000026222	ENSMUST00000153574
Sell	ENSMUSG00000026581	ENSMUST000000195358

Supplementary Figure 1

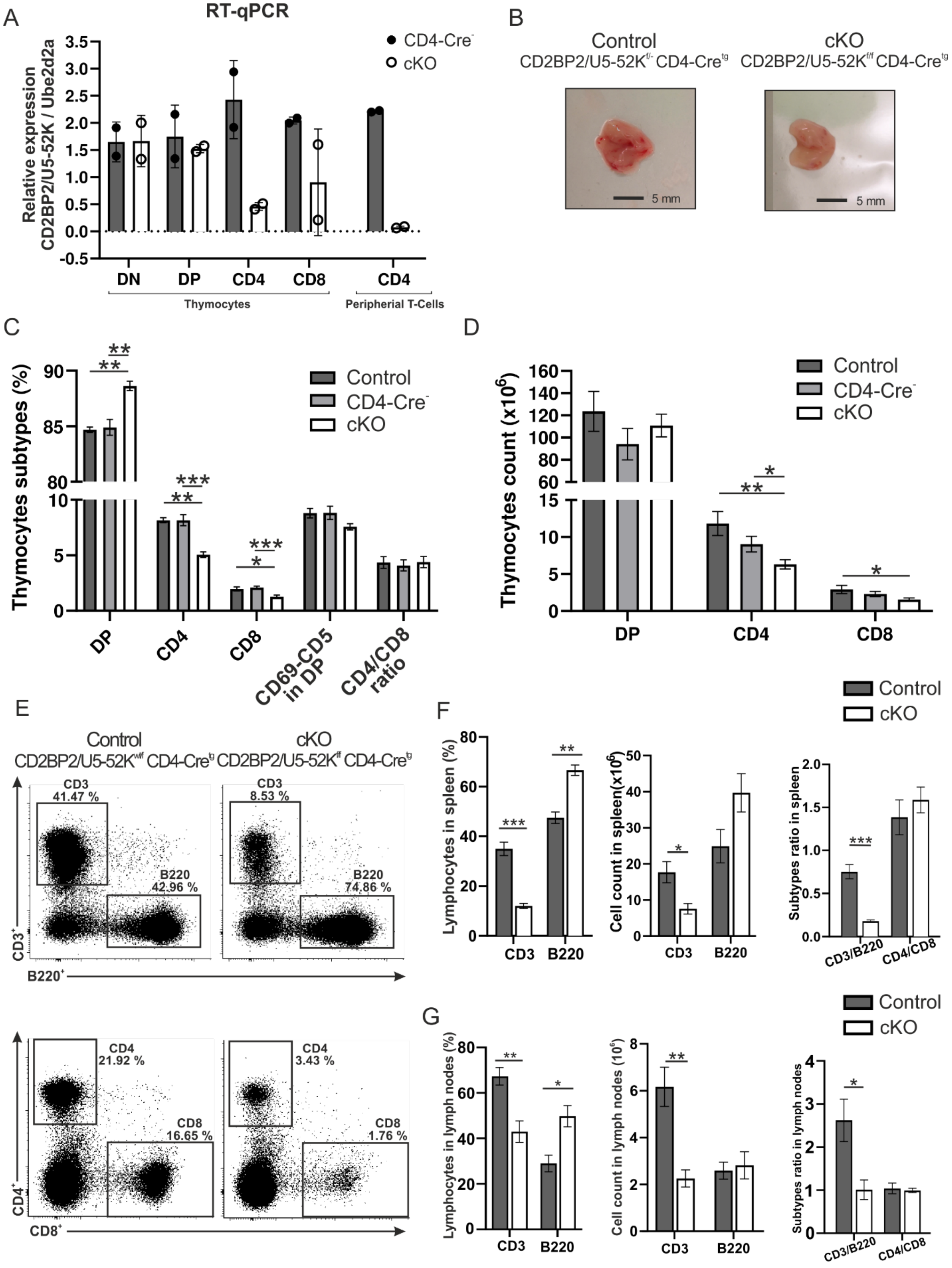

Suppl. Fig. 1 A) Analysis of *CD2BP2/U5-52K* gene and protein expression in CD2BP2/U5-52K$^{f/f}$ CD4-Cre^{tg} mice. A) *CD2BP2/U5-52K* gene expression analysis was performed by RT-qPCR. The RNA was extracted from thymocytes (DN, DP, CD4, CD8) and peripheral T cells (CD4) subtypes from CD2BP2/U5-52K$^{f/f}$ CD4-Cre^{tg} (cKO, n = 2) and CD2BP2/U5-52K$^{f/f}$ CD4-Cre^{-} (CD4-Cre^{-}, n = 2). The relative *CD2BP2/U5-52K* gene expression was analyzed in comparison with *Ube2d2* gene. B) Images of the thymi extracted from Control and cKO mice. C and D) Thymocytes analysis of CD2BP2/U5-52K$^{f/f}$ CD4-Cre^{tg} (cKO), CD2BP2/U5-52K$^{-/f}$ CD4-Cre^{tg} (Control) and CD2BP2/U5-52K$^{f/f}$ CD4-Cre^{-} (CD4-Cre^{-}). C) Bar diagram representing the percentage of DP, CD4, CD8, quadruple positive population (CD69^{+}, CD5^{+}, CD4^{+}, CD8^{+}) and CD4/CD8 ratio in Control (n = 6), CD4-Cre^{-} (n = 9), cKO (n = 8) mice. D) Bar diagram representing the absolute cell counts of the thymocyte subpopulations for Control (n = 5), CD4-Cre^{-} (n = 7) and cKO (n = 6). Bar represent mean ± SEM. Statistical analysis was performed using GraphPad Prism, using ANOVA one-way, unpaired Kruskal-Wallis test. p value description: $*p \leq 0.05$; $**p \leq 0.01$; $***p \leq 0.001$; no stars: not significant. E-H) Analysis of lymphocytes derived from spleen and peripheral lymph nodes. E) Representative flow cytometric dot plots of T cells from Control mouse (CD2BP2/U5-52K$^{wt/f}$ CD4-Cre^{tg}) and conditional KO (cKO) mouse (CD2BP2/U5-52K$^{f/f}$ CD4-Cre^{tg}). The dot plots show in the upper plots the CD3^{+} and B220^{+} populations and in the lower plot the double positive -CD4^{+} and the -CD8^{+} populations. F and G) Bar diagrams representing the percentage, the cell count and the T cell ratio of spleen (F) and lymph nodes (G). The bar diagrams (F-G) represent the mean of values detected in Control (n = 6) and cKO (n = 8) mice. The error bars represent the standard error (SEM). Statistical analysis was performed using GraphPad Prism, using non-parametric, unpaired Mann-Whitney test. p value description: $*p \leq 0.05$; $**p \leq 0.01$; $***p \leq 0.001$; no stars: not significant.

Supplementary figure 2

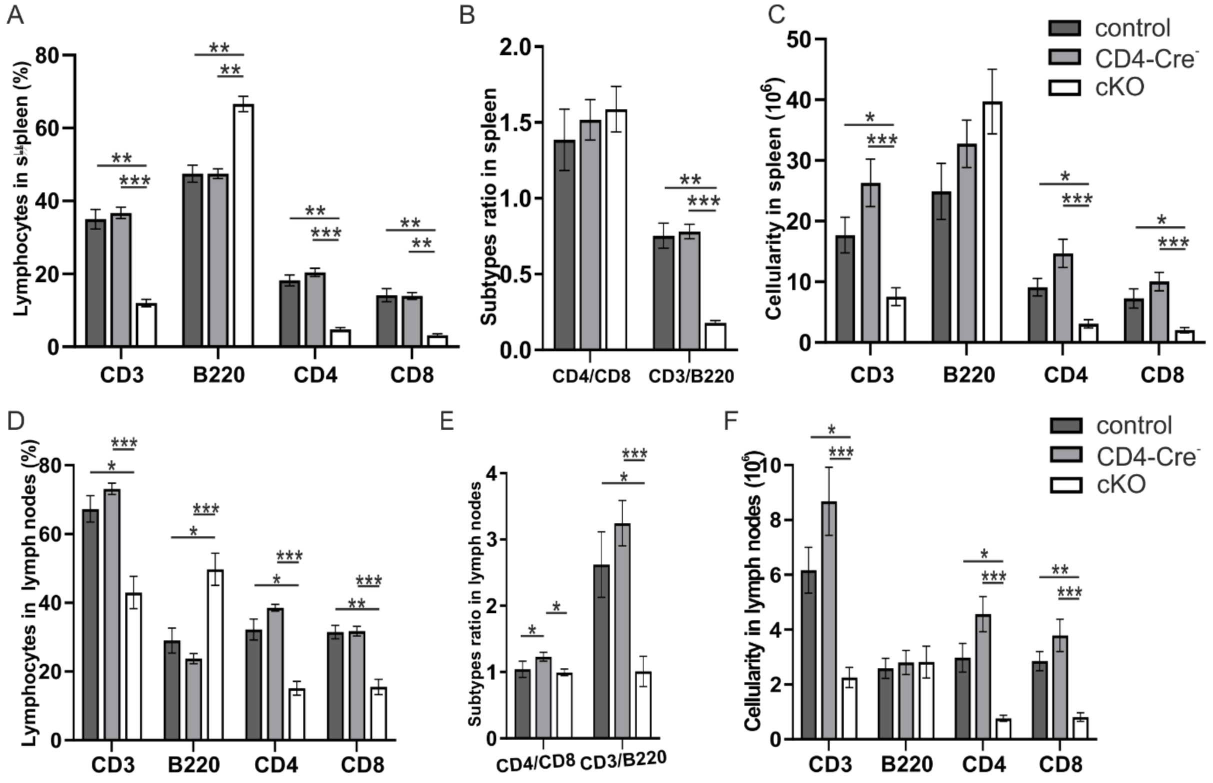

Suppl. Fig. 2 A and D) Bar diagrams representing the percentage of $CD3^{+}$, $B220^{+}$, $CD3^{+}$-$CD4^{+}$ and $CD3^{+}CD8^{+}$ populations in spleen (A) and peripheral lymph nodes (D). B and E) Bar diagrams representing the subtypes ratio population CD4/CD8 and CD3/B220 in spleen (B) and lymph nodes (E). C and F) Bar diagram representing the population cellularity in spleen (C) and lymph nodes (F). Each bar represents the mean value ± SEM detected for Control (CD2BP2/U5-$52K^{wt/f}$ CD4-Cre^{tg}, n = 5), CD4-Cre^{-} (CD2BP2/U5-$52K^{f/f}$ CD4-Cre^{-}, n = 9) and cKO (CD2BP2/U5-$52K^{f/f}$ CD4-Cre^{tg}, n = 8) mice. Statistical analysis was performed with GraphPad Prism, using ANOVA one-way, unpaired Kruskal-Wallis test. p value description: $^{*}p \leq 0.05$; $^{**}p \leq 0.01$; $^{***}p \leq 0.001$; no stars: not significant. The error bars represent the standard error (SEM). Statistical analysis was performed using GraphPad Prism, using non-parametric, unpaired Mann-Whitney test. p value description: $^{*}p \leq 0.05$; $^{**}p \leq 0.01$; $^{***}p \leq 0.001$; no stars: not significant.

Supplementary figure 3

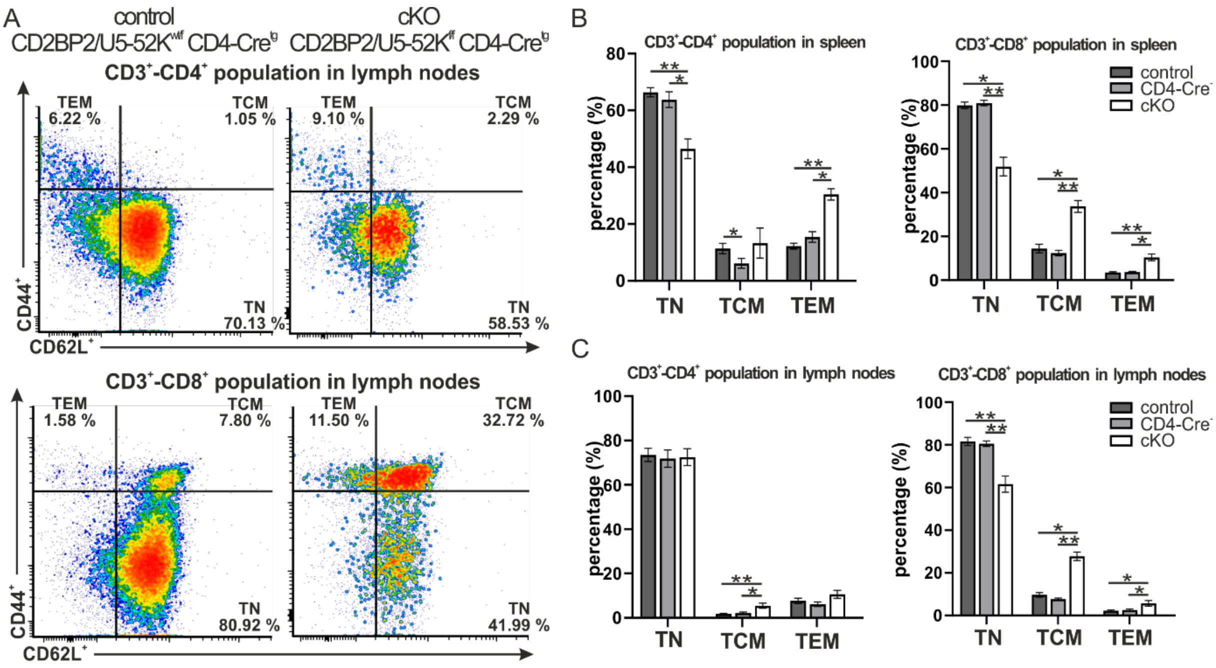

Suppl. Fig. 3 Naive and memory T cells analysis in spleen and peripheral lymph nodes. A) Representative flow cytometric dot plots of T cells from lymph nodes of Control (CD2BP2/U5-52K$^{f/f}$ CD4-Cre^{tg}) and cKO (CD2BP2/U5-52K$^{wt/f}$ CD4-Cre^{tg}) mice. The dot plots show naive and memory T cells using CD62L and CD44 markers for CD3^{+}-CD4^{+} (upper) and CD3^{+}-CD8^{+} (lower) populations. B and C) Bar diagrams representing the naïve T cells (TN), central memory T cells (TCM) and effector memory T cells (TEM) percentage in Control and cKO mice. The bar diagrams represent the percentage of naive and memory T cells in CD3^{+}-CD4^{+} and CD3^{+}-CD8^{+} population in spleen (B) and lymph nodes (C). All the bar diagrams represent the mean of values detected in Control (CD2BP2/U5-52K$^{wt/f}$ CD4-Cre^{tg}, n = 5-6), CD4-Cre^{-} (CD2BP2/U5-52K$^{f/f}$ CD4-Cre^{-}, n = 5) and cKO (CD2BP2/U5-52K$^{wt/f}$ CD4-Cre^{tg}, n = 5-6) mice. The error bars represent the standard error (SEM). Statistical analysis was performed with GraphPad Prism, using ANOVA one-way, unpaired Kruskal-Wallis test. p value description: *$p \leq 0.05$; **$p \leq 0.01$; ***$p \leq 0.001$; no stars: not significant.

Supplementary Fig. 4

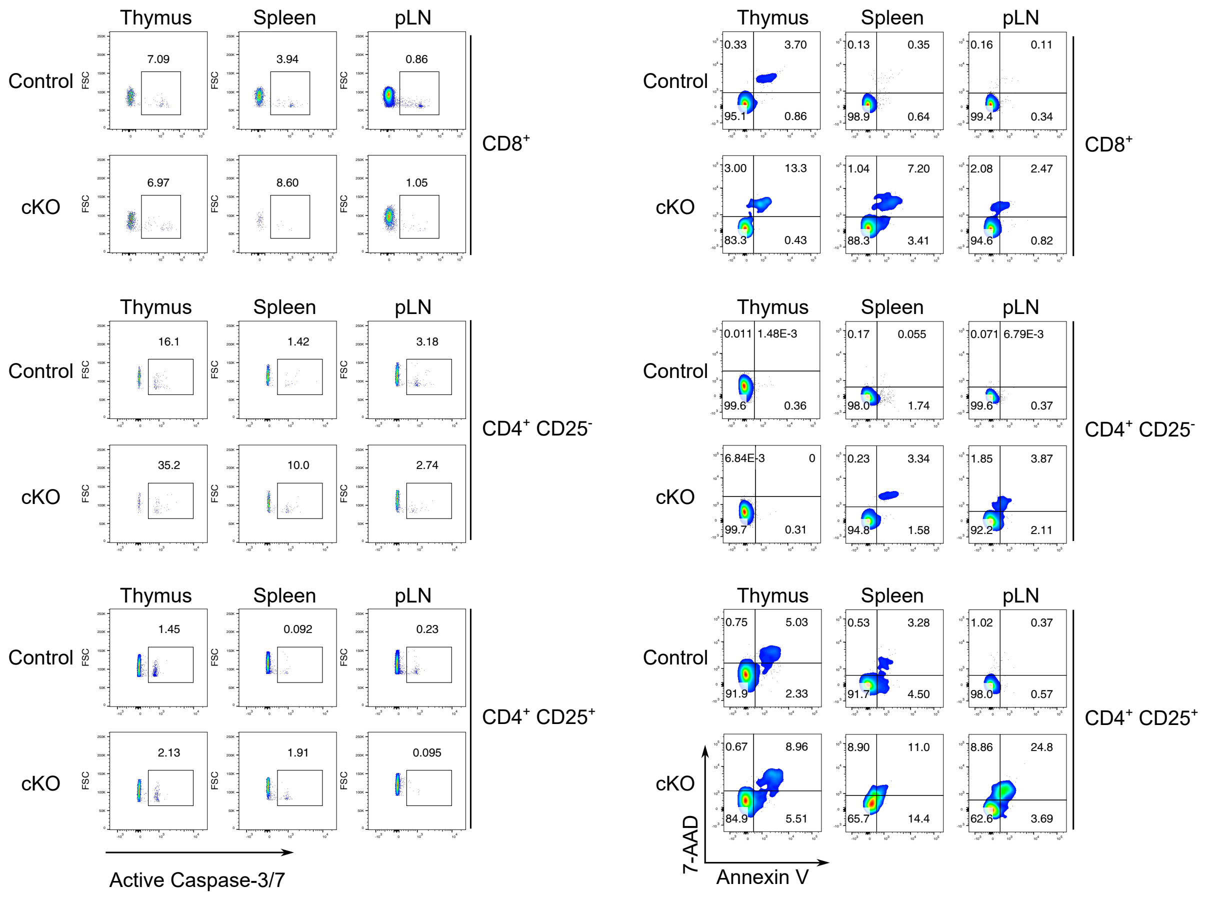

Supplementary Figure 4. T-cell apoptosis in the presence and absence of CD2BP2. (Left) Representative dot plots of active caspase-3/7-positive cells versus forward scatter (FSC). **(Right)** Representative dot plots of Annexin V versus 7-Aminoactinomycin D (7AAD)-positive cells. CD8+, Tconv (CD4+ CD25-) and CD4+ Treg (CD4+-CD25+) derived from thymus, spleen and peripheral lymph nodes (pLN) of control (CD2BP2/U5-52K$^{wt/f}$ CD4-Cre^{tg}) and conditional KO mice (CD2BP2/U5-52K$^{f/f}$ CD4-Cre^{tg}, cKO) were analyzed.

Supplementary Figure 5

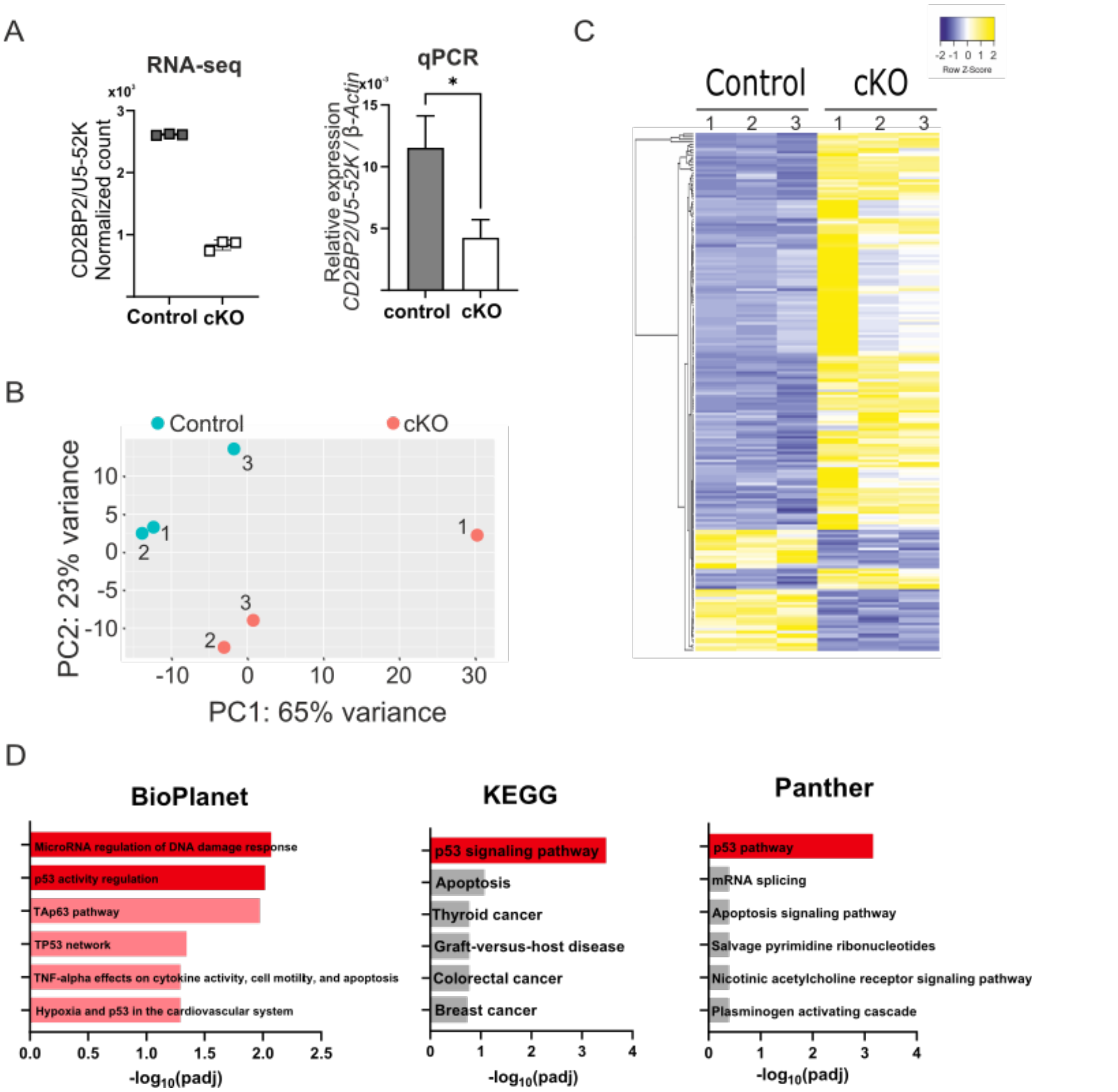

Suppl. Fig. 4 A) CD2BP2/U5-52K gene expression detected with RNA-seq analysis (left) and validate with RT-qPCR (right). For the validation, the RNA extracted from the $CD4^{+}$ T cells of 4 Control (CD2BP2/U5-$52K^{wt/f}$ CD4-Cre^{tg}) and 6 cKO (CD2BP2/U5-$52K^{f/f}$ CD4-Cre^{tg}) mice was used. The bar diagram represents the mean value and the errors bars represent the standard error (SEM). Statistical analysis was performed using GraphPad Prism, parametric unpaired t-test. p value description: $*p \leq 0.05$; $**p \leq 0.01$; $***p \leq 0.001$ and $****p \leq 0.0001$. B) Principal component analysis plot (PCA) describing the variance in the samples. The analysis of the 6 samples, 3 per each condition (CTRL in green and cKO in red), was calculated using DESeq2 software in R. C) Heatmap calculating the Eucledian distance between samples generated with all the significant DEGs using Heatmapper.ca. D) Enrichment pathway analysis performed with Enrichr program on BioPlanet, KEGG and Panther databases. The red bar indicates a padj < 0.01, the pink bar a padj between 0.05 and 0.01 and the gray is non-significant result.

Supplementary figure 6

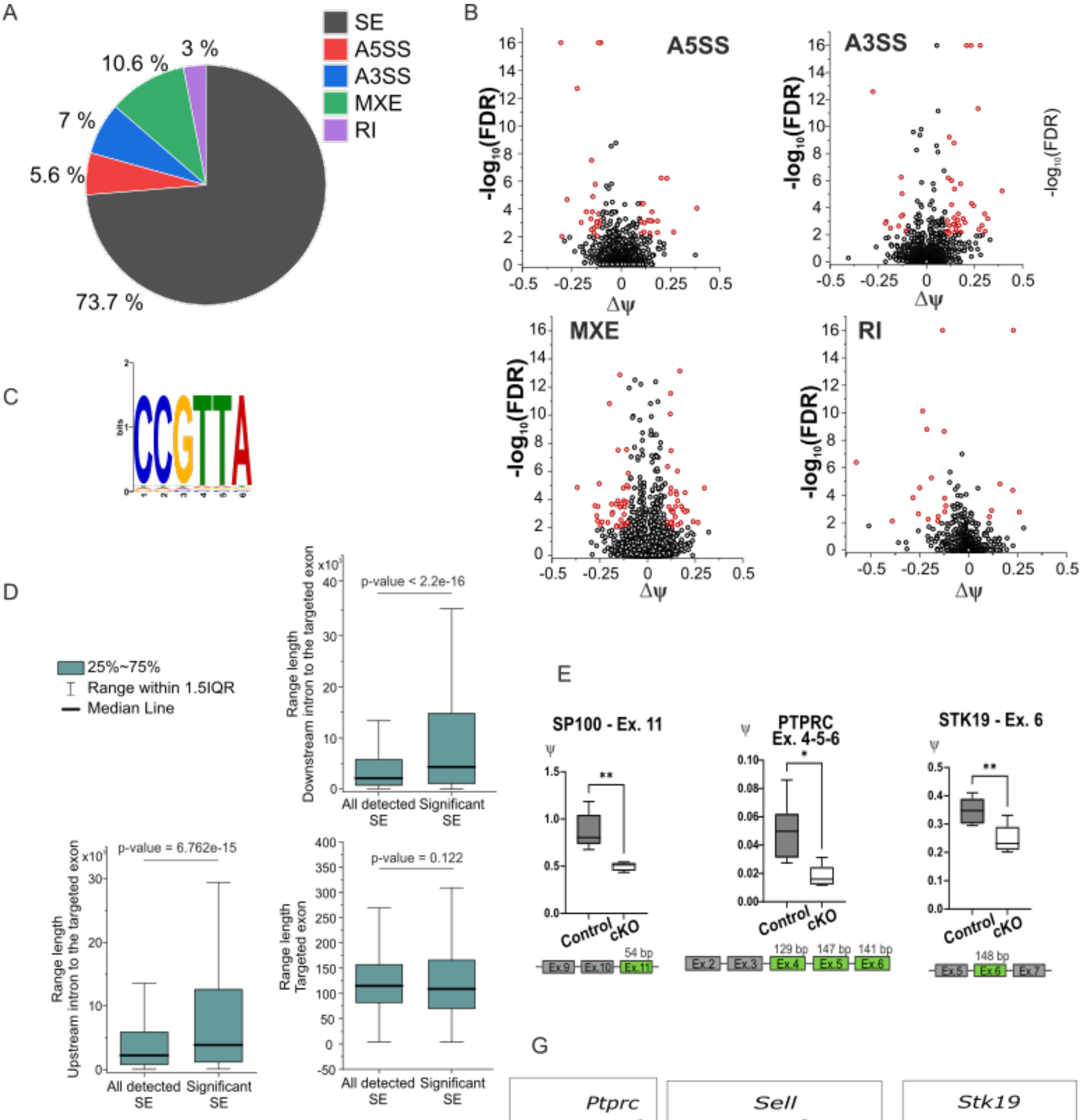

F

Gene	Number of cKO mice	Number of WT mice
MDM4	6	7
FOXN3	5	7
SELL	5	7
ARIH2	5	6
PTPRC	5	6
SP100	5	7
STK19	5	7

G

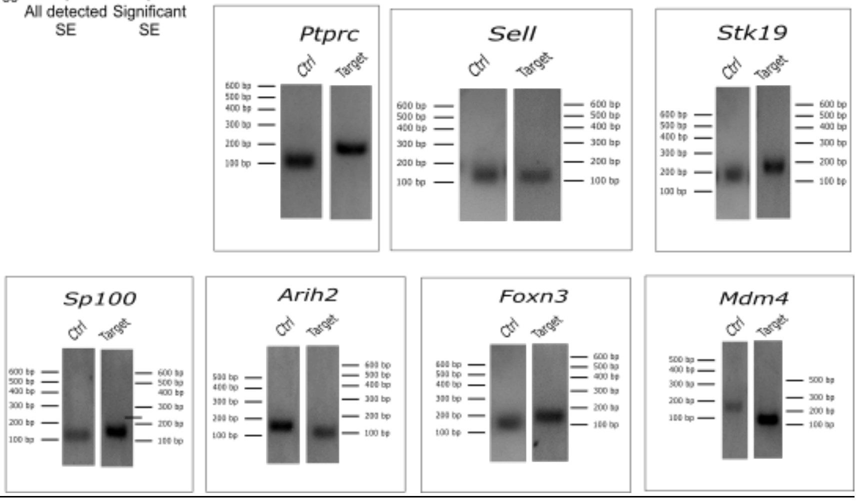

Suppl. Fig. 6 A) Pie chard representing the percentage of DSVs due to the lack of CD2BP2/U5-52K proteins. The splicing events are defined as Skipping exon (SE), Retain Intron (RI), Alternative 3' Splice Site (A3SS), Alternative 5' Splice Site (A5SS), Mutually Exclusive Exons (MXE). B) Volcano plot of the splicing events detected and selected based on the threshold count of RI, A5SS, A3SS and MXE. The red dots represent the DSVs that shows significant variance in the two conditions. C) Logo of the motif enriched in the sequences of the skipped exons. Analysis performed with STREME software. D) Analysis of the range length of introns and exons in the SE events detected, comparing all the detected SE with the significant SE. In the left the graph represents the length of upstream introns, in the middle the length of the targeted exon and the right the length of downstream intron ensuing the targeted exons. P-value was calculated with Wilcoxon test using R. E) Graphic representation of the validated skipping exon events of the gene *Ptprc*, *Sp100* and *Stk19*. In green are highlighted the exons that are alternatively spliced in cKO mice. F) Statistics on mice used for the data shown in Fig. 6 C and Suppl. Fig. 6 E. G) Gel electrophoresis of the amplicons after RT-qPCR for each of the seven validate targets (Figure 6 C and Suppl. Fig. 6 E.

Supplementary Figure 7

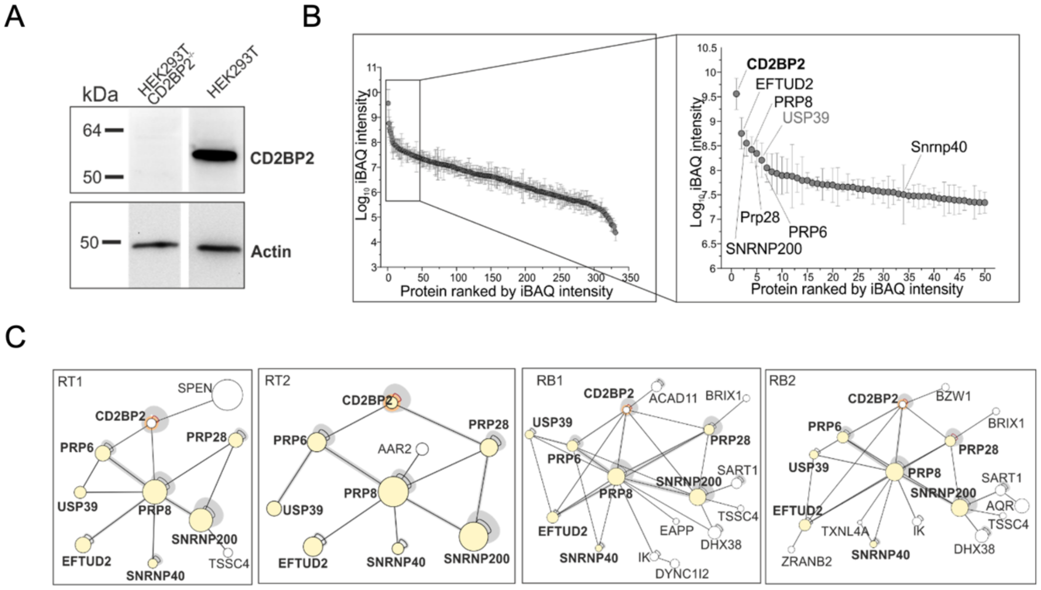

Supplementary Figure 7: A) Western blot representing the absence of CD2BP2/U5-52K protein in the KO cell line HEK293T CD2BP2/U5-52K $^{-/-}$ treated with CRISPR-Cas9. B) Ranking of all the identified protein from the IP by their relative intensity (iBAQ value). The data show that CD2BP2/U5-52K and protein form the U5-snRNP were highly abundant. C) Graphical representation of the crosslinked proteins identified from FLAG- CD2BP2/U5-52K immunoprecipitation followed by crosslinking and detection by mass spectrometry. The cross-linking data from the experiment, TR1, TR2, BR1 and BR2 were processed with xiNET software.

Supplementary figure 8

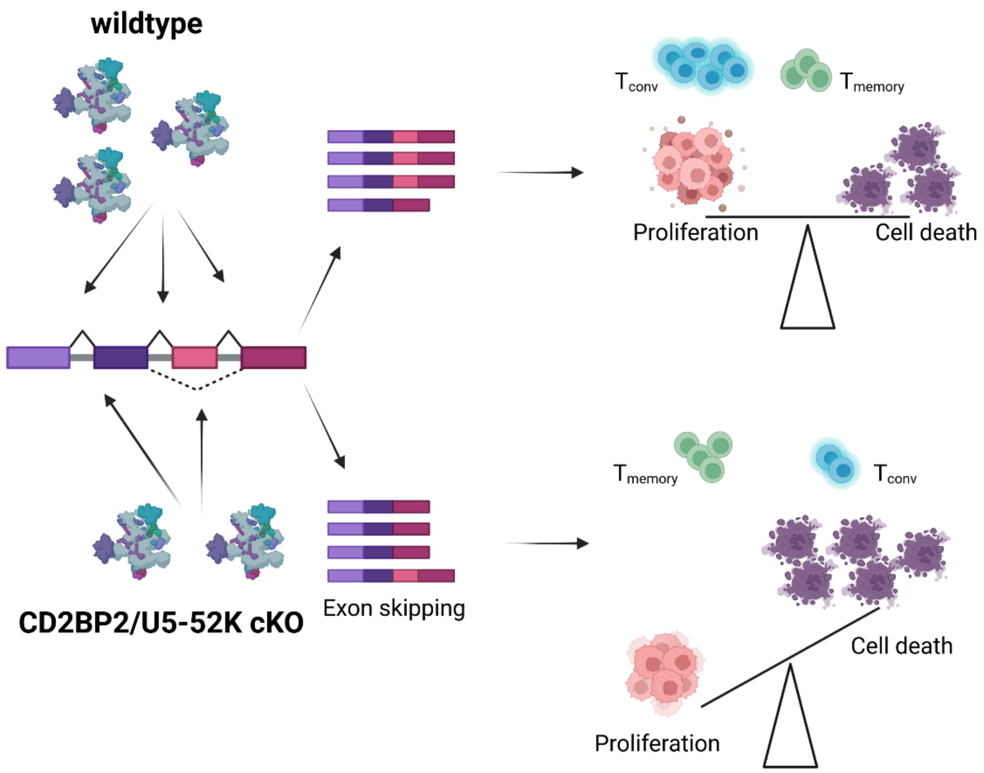

Suppl. fig. 8. Model of the role of CD2BP2/U5-52K for splicing efficiency. In the absence of CD2BP2/U5-52K splicing efficiency is reduced, probably due to a defect in U5 snRNP assembly, thus affecting all subsequent steps of the splicing cycle. As a consequence, exon skipping leads to increased expression of altered mRNA and protein isoforms or to NMD. This in turn has a dramatic effect on the differentiation/proliferation balance. In T cells, the altered homeostasis leads to a strong relative increase of memory T cells compared to naïve and effector T cells
